# Topological dynamics of an intrinsically disordered N‐terminal domain of the human androgen receptor

**DOI:** 10.1002/pro.4334

**Published:** 2022-05-26

**Authors:** Vahid Sheikhhassani, Barbara Scalvini, Julian Ng, Laurens W. H. J. Heling, Yosri Ayache, Tom M. J. Evers, Eva Estébanez‐Perpiñá, Iain J. McEwan, Alireza Mashaghi

**Affiliations:** ^1^ Medical Systems Biophysics and Bioengineering, Leiden Academic Centre for Drug Research, Faculty of Science Leiden University Leiden The Netherlands; ^2^ Centre for Interdisciplinary Genome Research, Faculty of Science Leiden University Leiden The Netherlands; ^3^ Department of Biochemistry and Molecular Biomedicine Institute of Biomedicine (IBUB) of the University of Barcelona (UB) Barcelona Spain; ^4^ Institute of Medical Sciences, School of Medicine, Medical Sciences and Nutrition, University of Aberdeen Scotland UK

**Keywords:** androgen receptor, circuit topology, conformational dynamics, intrachain contacts, nuclear hormone receptors

## Abstract

Human androgen receptor contains a large N‐terminal domain (AR‐NTD) that is highly dynamic and this poses a major challenge for experimental and computational analysis to decipher its conformation. Misfolding of the AR‐NTD is implicated in prostate cancer and Kennedy's disease, yet our knowledge of its structure is limited to primary sequence information of the chain and a few functionally important secondary structure motifs. Here, we employed an innovative combination of molecular dynamics simulations and circuit topology (CT) analysis to identify the tertiary structure of AR‐NTD. We found that the AR‐NTD adopts highly dynamic loopy conformations with two identifiable regions with distinct topological make‐up and dynamics. This consists of a N‐terminal region (NR, residues 1–224) and a C‐terminal region (CR, residues 225–538), which carries a dense core. Topological mapping of the dynamics reveals a traceable time‐scale dependent topological evolution. NR adopts different positioning with respect to the CR and forms a cleft that can partly enclose the hormone‐bound ligand‐binding domain (LBD) of the androgen receptor. Furthermore, our data suggest a model in which dynamic NR and CR compete for binding to the DNA‐binding domain of the receptor, thereby regulating the accessibility of its DNA‐binding site. Our approach allowed for the identification of a previously unknown regulatory binding site within the CR core, revealing the structural mechanisms of action of AR inhibitor EPI‐001, and paving the way for other drug discovery applications.

## 1 INTRODUCTION

A key structural element of nuclear hormone receptors (NHRs) is the N‐terminal transactivation domain (NTD), which is critical for receptor function, yet its structure is unknown. This is due to its intrinsic disorder and high dynamics that prevent its structural determination. As such, molecular mechanism behind NTD function remains elusive.^1^, ^2^, ^3^ In the case of the androgen receptor (AR), recent cryo‐electron microscopy (cryo‐EM) analysis of the full‐length receptor in complex with an interacting coregulatory partner, suggest that the NTD is a disordered conformation that surrounds the ligand‐binding domain (LBD).^4^ However, additional studies show the AR‐NTD can also fold in functional state, as truncated forms of AR, devoid of the LBD, act as constitutively fully active form,^5^ suggesting a central role of the NTD as a transcriptional driver.^6^, ^7^ This makes the NTD an interesting target for drug development,^8^, ^9^, ^10^ however the absence of high‐resolution information has challenged this development.

Studying the conformation of highly dynamic intrinsically disordered protein regions (IDRs) is a major challenge in structural biology.^11^, ^12^ Some important steps have been taken including insights from single‐molecule fluorescence resonance energy transfer (smFRET), electron paramagnetic resonance (EPR), and nuclear magnetic resonance (NMR).^13^ However, none have been applied successfully to resolve the structure of the full‐length AR‐NTD due to technical difficulties.^14^, ^15^, ^16^

The intrinsic disorder poses a challenge not only for experimental analysis of the conformation but also for computational modeling of the chain due to the size of the conformation space and lack of stable folds. For example, the state‐of‐the art artificial intelligence‐based prediction approaches fail to identify the conformation of AR‐NTD.^17^ As such, new technological innovations are needed to study intrinsically disordered protein chains. Despite being disordered, it is reasonable to assume that at least some residues in the disordered protein regions will adopt certain arrangements to function.^18^ Topological analysis might be able to capture those invariant arrangements hidden within a dynamic geometry. However, the field of protein topology has been primarily focused on knotted conformations and ignored intra chain contacts,^19^, ^20^ and the field of structural biology mainly concentrated on geometric analysis of protein chains. Circuit topology (CT) has recently emerged as a complementary topology approach to knot theory^21^, ^22^ and is able to fully characterize folded molecular chains.^23^, ^24^ Circuit topology is a theoretical framework which only focuses on intra‐chain contacts and their arrangement within the chain. If we pick any pair of contacts in the chain, their topological relation can either be one of three possible arrangements: series (S), parallel (P), and cross (X) (Figure S1). Contacts which are in series do not intersect and belong to different sub‐sections of the chain. Parallel relation implies that the first contact is formed by a sub‐section of the chain included in the two contact sites of the second contact. Two contacts in cross relation intersect but belong to a different class with respect to parallel relations, since the contact sites of one loop do not fully encompass the other. One can establish the topological relation of any two contacts in the chain: the total number of S, P, and X can be used as the simplest measure of the topological state of the chain. The CT approach has led to unprecedented insights into the role of topology in molecular folding processes.^25^, ^26^, ^27^, ^28^, ^29^, ^30^, ^31^, ^32^, ^33^, ^34^ However, circuit topology has not yet been used to study intrinsically disordered proteins, and thus the topology of IDPs/IDRs is an unexplored area.

Here, we combine an innovative CT‐based analysis with molecular dynamics (MD) simulations to study an intrinsically disordered protein chain for the first time. We focus on the AR‐NTD, given its key role in receptor physiology and mediating diseases.^35^, ^36^, ^37^ We search for topological patterns and investigate their implications for AR receptor assembly, target DNA binding, and interaction with AR inhibitor drugs.

## 2 METHODS

### 2.1 Modeling of three‐dimensional structure of AR‐NTD

Due to its disordered conformation, there is no resolved structure of the NTD domain deposited on the Protein Data Bank (PDB). To start from a computationally efficient initial structure, I‐TASSER server^38^ was used for modeling the three‐dimensional structure of the AR‐NTD (residues 1–538). I‐TASSER is a hierarchical approach to protein structure prediction and structure‐based function annotation. It is ranked the best protein structure prediction method by the Critical Assessment of Protein Structure Prediction (CASP) community. The *Z*‐score and normalized *Z*‐score >1 indicates a template with a good alignment quality. The Confidence‐score (*C*‐score, values −5 to 2) reflects the threading alignment and convergence of structure refinement simulations with higher score corresponding to a high confidence model. A Template Modeling (TM) score >0.5 indicates correct conformation of the model, while scores <0.17 corresponds to a model of random topology. Therefore, models with the highest *Z*‐score, *C*‐score and TM‐score were selected for our MD studies.

In order to further optimize and validate the initial structure, an energy minimization step using steepest descent method was performed followed by a conjugate gradient with a ff99SB all atom force field to perform a total of 100,000 steps using the GROMACS software package.^39^ For validation of energy refined structures, WHATIF^40^ was used for anomalous bond angle and bond length detection. RAMPAGE^41^ was used for Ramachandran plot analysis and detection of incorrectly modeled protein regions was done using ERRAT.^30^ The compatibility of the three‐dimensional protein model to amino acid sequence was verified using VERIFY3D^42^ and the global quality of the models was calculated using QMEAN.^43^ Structural disorder was analyzed using the PONDR web server and plots directly obtained from the server.^44^

### 2.2 Molecular dynamics setup

In this study, 5 μs of MD simulations was carried out using the SIRAH coarse‐grained force field for proteins in combination with the WT4 explicit coarse‐grained water model.^45^ The advantage of using this model is to reproduce the roughly tetrahedral ordering of bulk water due to the existence of hydrogen bonds between atomistic water molecules. Three MD simulation with different random seeds started from energy minimized predicted structure obtained from structure prediction pipeline. The entire AR‐NTD (residues 1–538) was mapped to a coarse‐grained representation according to the standard SIRAH mapping. Rhombic dodecahedron box was used to dissolve NTD structure by adding WT4 water molecules. Electroneutrality and physiological concentration of salt were achieved by replacing corresponding amount of water molecules with NaW and ClW (coarse‐grained representations of Na+ and Cl− ions, respectively). All systems were minimized using the steepest descent algorithm and then went through a 5 ns NVT equilibration, a 5 ns NPT equilibration, and a NPT production run. The leapfrog integrator with a 20 fs time step was used throughout. Protein beads were constrained with the LINCS algorithm^46^ during the equilibration, and no constraints were used during the minimization and production. The temperature was kept at 310 K with a velocity rescale thermostat,^47^ and the pressure at 1 bar with the Parrinello−Rahman barostat.^48^ τ_T_ for the thermostat was set to 1.0 ps during the equilibration phases and to 2.0 ps during the production. τ_P_ for the barostat was set to 10.0 ps during both the NPT equilibration and the production. Both nonbonded cut‐offs (van der Waals and shortrange electrostatics) were set to 1.2 nm. Long‐range electrostatics were treated with the PME method with a 0.2 nm grid spacing during the equilibration and 0.25 nm during the production. Nonbonded interactions were calculated using a 1.2 nm cut‐off neighbor list, updated every 25 steps (in the production and the NPT equilibration) or 10 steps (in the NVT equilibration). Both energy and pressure dispersion corrections were applied. Periodic boundary conditions and the minimum image convention were used. Snapshots were collected every 1,000 steps (20 ps). All simulations and analyses were carried out with the GROMACS 2020 software.^39^

### 2.3 Molecular docking

The binding interactions of NTD with LBD and DBD was studied using the ClusPro web server^49^ with the aim of identifying interaction sites. This docking suite has been consistently rated among the best global docking procedures in the CAPRI challenge (Critical Assessment of Predicted Interaction).^49^ ClusPro first performs rigid body docking by sampling a wide range of conformations and subsequently clusters the thousand lowest‐energy structures based on root‐mean‐square deviation (RMSD) to identify the largest clusters that represent the most likely models. It furthermore refines the represented structures by energy minimizing the CHARMM potential.^49^ We supplied ClusPro with previously reported crystal structures for DBD (PDB code: 1R4I), LBD (PDB code: 1T7T), and RAP74‐CTD (PDB code: 1I27) to dock with our energy minimized structure of the NTD. The top 10 best ranked models in ClusPro were selected for further analysis.

In order to find the possible binding site of the EPI‐001 compound on the CR region of the NTD, drug–protein interaction analysis was performed. The mol file of the EPI‐001 was created in online molecular drawing module and then transferred to PRODRG server^50^ to generate the required topologies and structure coordinates. The representative structures of three clusters obtained from clustering the CR region of NTD were prepared for docking by adding Kollman charges and hydrogen atoms to the polar groups of the protein using AutoDock tools.^51^ AutoDock 4.2 package^51^ was used for docking the EPI‐001 to the target molecules. Blind docking was performed for the drug using the default parameters on Autodock.

### 2.4 Preparing the structures for circuit topology analysis

After the trajectory of the system was obtained, atomic positions of amino acids were generated from the location of CG beads. Backmapping was done using sirah_vmdtk.tcl plugin followed by 100 steps of steepest‐descent and 50 steps of Conjugated Gradient minimization in vacuum using the sander module of AmberTools.^42^ This process made the procedure robust and independent from the fine details of the backmapping library. The obtained atomistic coordinates were used for circuit topology analysis.

### 2.5 Circuit topology analysis

Circuit topology parameters were retrieved using custom‐made Python code, modified for energy, length filtering, and circuit decomposition. For doing this, 1,000 frames were extracted from the trajectory containing only the protein molecule. Contacts between residues were calculated based on a distance cut‐off of 4.5 Å. Residue pairs where more than five atoms were found to be at a respective distance below the distance cut‐off were computed as contacts. The three closest neighbors of each residue were excluded from analysis.

In order to perform dynamic CT analysis, frames from the AR‐NTD simulations were taken 5 ns apart and transformed into binary contact maps. The stack of contact maps was processed to create the contact life‐time kymographs: all combinations of residues which never participate in contacts were removed. In order to create the contact lifetime distribution, the maximum lifetime was chosen for each particular combination of residues.

Time filtering for topological study was performed by creating life‐time filters matrices. These matrices were then multiplied to regular contact matrices as a customization of the CT Python tool. All dynamic CT analysis was performed using a custom‐made Jupyter lab data analysis file.

Accession numbers:

PDB: 1R4I, 1T7T, 1I27.

## 3 RESULTS AND DISCUSSIONS

### 3.1 AR‐NTD folds into two disjoint highly dynamic regions

**FIGURE 1:**
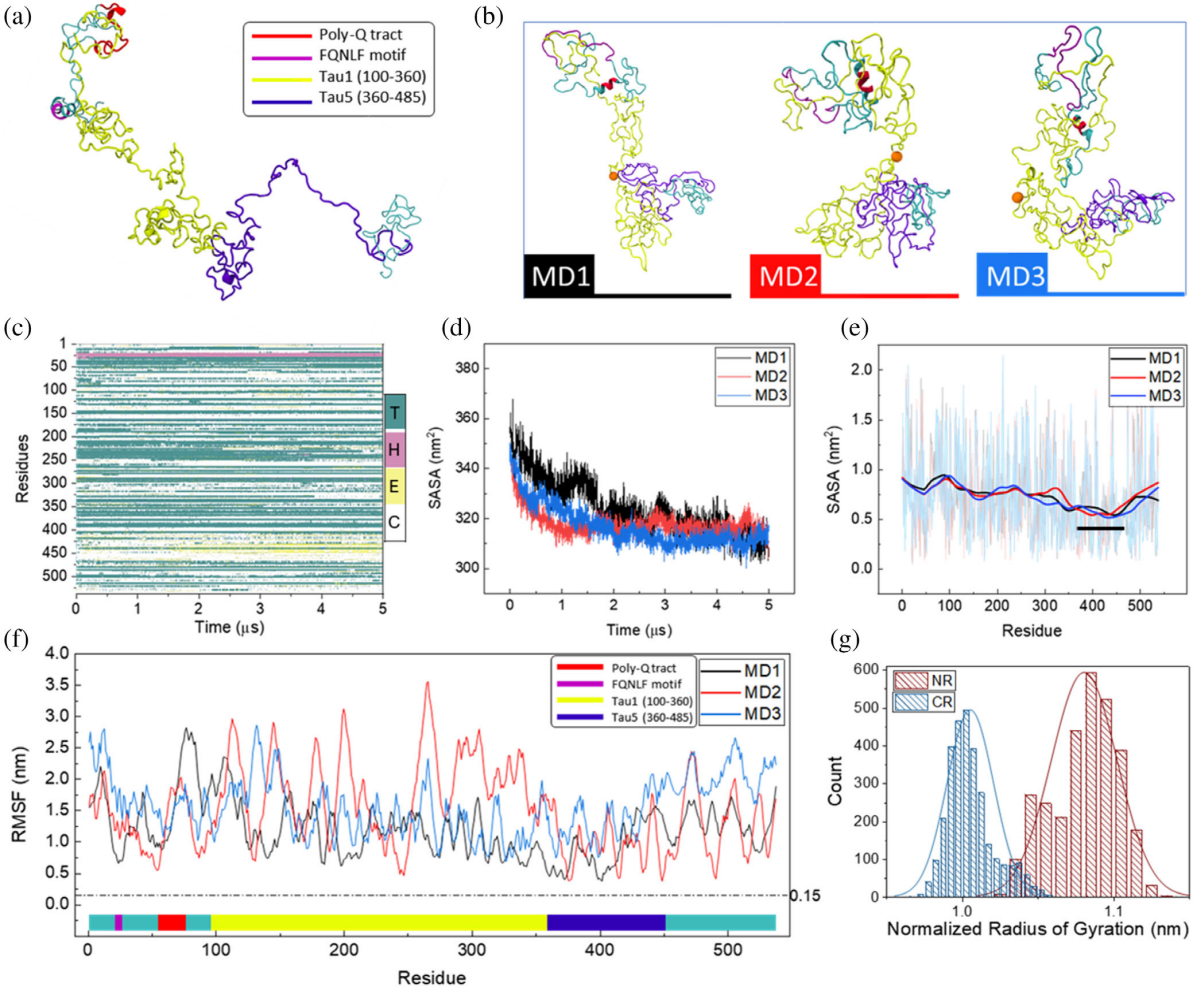
(a) Initial structure of the AR‐NTD, cartoon representation is colored based on different functional motifs including PolyQ (residues 58–80) which is colored in red, FQNLF (residues 23–27) in purple, Trans activation unit 1 (Tau‐1, residues 100–360) in yellow, and Trans activation unit 5 (Tau‐5, residues 360–485) in blue. (b) three representative conformations from the last 10 ns of each replicate of the MD simulations. (c) Secondary structure evolution of NTD during 5 μs of simulation. With T, H, E, and C representing Turns, Helix, Strand, and Random Coil, respectively. (d) Time evolution of the solvent accessible surface area (SASA) of NTD. For all three replicates of the simulation, SASA values were decreased due to the overall compaction of the structure. (e) Solvent accessible surface area calculated for each residue during the last 3 μs of the simulation. Small black line indicates CR core (residues 355–469). (f) Average root mean square fluctuations of AR‐NTD calculated per residue over the last 3 μs of the simulation. The dashed line indicates the average RMSF of folded AR‐LBD (0.15 nm). (g) The size‐normalized radius of the CR and NR regions during the last 3 μs of the simulation.

First, we performed molecular dynamics simulations of the AR‐NTD in an aqueous solution with physiological salt concentration. The initial structure, shown in Figure 1a, was built using I‐TASSER server.^38^ The model was chosen as the best ranked model obtained from the server. The model was superior to conformations predicted by the AlphaFold and RosettaFold severs, based on confidence measures and comparative analysis with experimental data (Figure S2). Comparison between α‐helix content of the models predicted by different algorithms with Circular Dichroism analysis of the NTD^18^ revealed that among the predicted models, the best ranked model from I‐TASSER and the AlphaFold model showed closer values to CD data. However, given the very low confidence level of the AlphaFold model, the best ranked model obtained from I‐TASSER was chosen for performing MD simulations.

After minimization and relaxation, we performed MD simulations of the NTD structure. Visual examination of the trajectory and RMSD plots show that the initial conformation has undergone an extensive structural change (Figures S3 a and 1b). Figure 1c provides a kymograph of all the secondary structures within the NTD, which is mostly composed of random coils, turns, and loops despite the apparent changes in the global 3D shape of the system (Figure S3 b and c). We repeated the MD simulation three times using different initial velocities to ensure that our observations are not dependent on the initial conditions. Importantly, all three independent runs consistently led to the emergence of two disjoint regions in 3D within 2 μs of simulations: an extended N‐terminal sub‐region (NR, residues 1–224), and a C‐terminal sub‐region (CR, residues 225–538). Both sub‐regions showed high level disorder and high solvent accessibility, which reached a plateau after 2 μs of simulations (Figure 1d,e). We monitored the dynamics for an additional 3 μs and computed root‐mean‐square fluctuations (RMSF) to quantify the fluctuations of the chain. We observed an order of magnitude larger RMSF for AR‐NTD in comparison to the folded AR‐LBD (Figure 1f, dashed line at 0.15 nm). Considering the average fluctuations of the chain we observed lower values of RMSF in the vicinity of residue 400. Quantification of solvent accessibility revealed that both CR and NR are highly solvent accessible. The CR however has a central segment (residues 355–469) which is relatively less solvent accessible (Figures 1e and S4), corresponding to the reduction seen in the RMSF profile. Disorder prediction data produced by PONDR analysis agrees with the solvent accessibility and RMSF profiles, with the central region within CR having less disorder than the rest of the CR (Figure S5). We denote this segment as *CR core*, to differentiate it from the rest of the CR, *CR shell*. Interestingly the core includes the well‐known transcriptional activation unit 5 (Tau‐5) motif of the AR‐NTD. Comparing the radii of gyration of CR and NR, one can clearly see that the CR is significantly more compact than NR (Figure 1g). This is consistent with our model that CR, despite its disorder, carries residual structures in its core.

To further demonstrate that the formation of disjoint regions does not depend on the choice of initial configurations, we melted the compact NTD at 350 K and then ran the simulation for 1 μs at physiological temperature. Melting opened up the compact structures and led to disappearance of separation between regions. The subsequent simulations, however, recovered the NR/CR in all three experiments and the low solvent accessibility of the CR core. Moreover, we find that our observation of NTD folding to two disjoint regions does not depend on the *F*‐parameter of the force field, which encodes the interactions between the polypeptide and the surrounding solvent (Figure S6). We note that compaction of the chain to form the CR/NR segregation and the emergence of the CR core are AR‐NTD specific, as synthetic models of AR‐NTD using large poly‐glutamine (polyQ) or poly‐alanine (polyA) stretches, generated by mutating all residues of the structure shown in Figure 1a to Q or A respectively, did not produce these features (Figure S7, S8).

It is noteworthy that we have used these two models as control analysis to better understand the complex behavior of the AR‐NTD (particularly during the first 1 μs). The synthetic polyQ model used to demonstrate the sequence specificity is not to be confused with a natural short polyQ motif that is embedded within the AR‐NTD. Q and A residues are known as disorder promoting and order promoting amino acids and were thus chosen to build these synthetic models with extreme behaviours. Although it has recently been shown that short lengths of the polyQ tract (up to 25 residues) can form a stable alpha helical structure^14^ there is evidence that extended lengths of the polyQ chain show a largely disorderd structure,^52^ while polyA shows a high propensity to form ordered structures.^53^

### 3.2 Mapping CR folding dynamics to the topology landscape showed a directional conformational evolution

A natural way to express how the NTD peptide chain folds dynamically is to describe its topological folding patterns over time. In contrast to the conventional geometric approaches, topological approaches are specifically designed to quantify and categorize residual structural (shape) properties of constantly deforming polypeptides. Thus, we decided to map the chain dynamics onto the chain topology space, to identify patterns or motifs of the folding process.^54^ Contacts in the chain are defined based on the spatial proximity of residues. The arrangement of residue–residue contacts provides a first‐order topological representation of a folded chain, which can be highly informative on folding processes.^25^ Here, we [46] exploited the CT framework to provide a dynamic topological description of a disordered protein for the first time. In order to map the topological dynamics of the NTD over time, we calculated the percentages of P, S, and X in chain conformations at 5 ns time points with a total of 1,000 frames over 5 μs. In this way, we followed the topological dynamics of the chain in time (Figure S9).

The topological analysis revealed critical differences in the folding process of NR and CR. While a clear topological trajectory is present for CR, the NR topology map is not showing any readily visible pathway. As seen in Figure S9 c, the starting conformation of CR was rich in series (S) but low in parallel (P) and cross (X) configurations. However, over time, while the percentage of P was almost constant, major changes were observed for S and X content, over all three runs. This topological profile is consistent with the observed emergence of the CR core. Ideally, an unfolded or semi‐unfolded chain will present local contacts at first,^55^ which have little interaction with far away residues along the chain. Non‐interacting contacts belong by definition to the S topological configuration. However, as the folding process develops, the chain will fold onto itself, creating long‐range interactions between residues, with a more complex topological profile. This process is true for any folding process, as exemplified by the topological evolution of our polyQ model of AR‐NTD, see Methods section for details about the polyQ model.

In contrast to the CR topological trajectory, the NR region explores the topological space in what appears to be a random fashion. As expected from such a highly dynamic region, the opportunities of forming and breaking contacts with high frequency leads to a very wide configurational space. This results in a globular pattern in the topological space. The lack of a preferred direction for topological evolution is nicely visualized by the width and overall shape of the pattern. This is in contrast with the narrow topological evolution of CR, which indicates a number of transient states with significantly different configurations: once a new configuration is reached, the set of possible contacts changes. This phenomenon can be visualized on the topological space as a “leap” from one cluster to another, resulting in a narrow trajectory with high directionality. The lower percentage of series relations relative to the CR indicates that the NR evolution is truly dynamic in nature, with long‐range contacts continuously forming and breaking and the absence of sub‐structures. Indeed, contacts internal to a sub‐structure are most likely in series with contacts embedded into another sub‐structure. This motivated us to look more closely into the topological dynamics of NR and search for patterns using our circuit topology approach.

### 3.3 Dynamic CT analysis reveals temporal regimes for topological development

**FIGURE 2:**
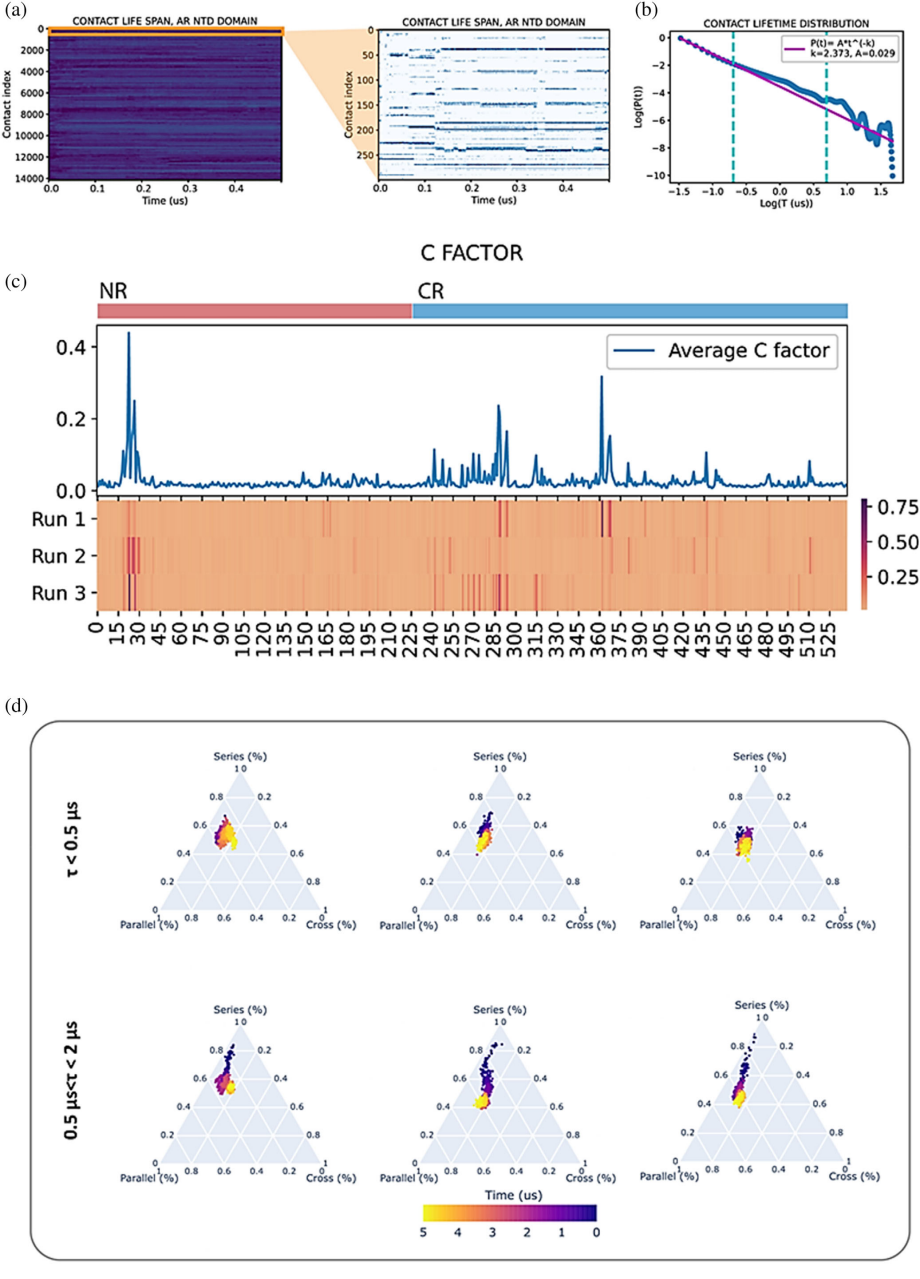
(a) Contact lifetime analysis reveals temporal regimes for topological evolution. A NTD contact lifetime kymograph, with zoomed detail of the first 300 contacts. (b) Log–log plot distribution of contact lifetime (maximum lifetime was selected for each contact) with power law fit. The dashed lines indicate the thresholds delimiting the three regimes: short, middle, and long lifetime. (c) Heatmap of C factor (average contact lifetime per residue) for all three runs, with plot of the C factor averaged over the three runs. (d) Triangular plots of the S/P/X topological space for NR, over all three runs, filtered for short and middle life contacts.

Dynamic topological analysis of the AR‐NTD shows the presence of three different time regimes for topological evolution, characterized by the time scales of residue–residue contact lifetimes. We envisioned a dynamic CT‐based topological analysis to identify the time scale of topological evolution of the two previously identified NTD sub‐regions, NR and CR. This, it is possible to follow the temporal evolution of every residue–residue contact formed at any moment of the MD simulation by plotting a kymograph of all possible residue‐residue contact combinations (Figure 2a). By looking at the kymograph (and zooming in on regions of interest), we see how some contacts are fairly short‐lived, often breaking and reforming with high frequency. On the other hand, other contacts live for a few microseconds. Therefore, we might have one (or more) lifetimes for each residue–residue contact: by picking the maximum lifetime of each particular residue combination, we can build a lifetime distribution of all contacts (Figure 2b).

As expected by such a dynamic and heterogeneous system, contact lifetimes span across the whole range of the simulation, with the vast majority of them being short lived (lifetime distribution maxima are 35, 18, and 15 ns, respectively for the three runs). The distribution in Figure 2b respects to some extent the well‐known scale free law ($P\left(\tau \right)=A\tau^{-\gamma }$), indicating there might not be a preferential duration for contact lifetimes.^56^ We see, however, that the degree with which the lifetime data match the power law is not the same for all time scales: we identify a “short life” range (τ<0.5μs), where matching is maximum. After 0.5 μs the distribution starts deviating from the scale‐free behavior (0.5μs<τ<2μs, “middle life”). After 2 μs, instability prevails, and deviations from the law appear with wide fluctuations, probably due to poor fitting of the distribution caused by the lack of statistics. The scale free distribution might represent a useful ideal model to quantify the behavior of IDPs. The adherence to this law is to a somewhat higher degree present for our polyQ case study (Figure S10). This regime behavior is highly conserved over all three runs, for both NR and CR (Figure S11). We can calculate the C‐factor for each residue, that is, the average contact lifetime among all contacts in which the residue participates (Figures 2c, S12,13). Here, this heterogeneity becomes apparent. If we exclude residue 23–27, where the only alpha helix present within the NTD resides, higher lifetimes are way more prevalent in the CR region, indicating higher stability and compaction with respect to the NR region. This stark difference in behavior between the two regions cannot be replicated in the synthetic polyQ (Figure S10), indicating this asymmetry is specific to AR‐NTD.

The lifetime analysis allows the identification of connectivity properties within the NTD under three different time regimes. This information can be exploited to disentangle the role of fast forming, short‐lived contacts from that of longer‐lived contacts during topological evolution. Next, we applied a lifetime filter to NTD contact maps and repeated the topological analysis. Results for short and middle life NR contacts can be seen in Figure 2d. Short life contacts seem overall to occupy only one topological state, over all the simulation, which is represented as a globule in the triangular S/P/X topological space. While these contacts oscillate around what looks like a wide topological equilibrium state, with no preferred direction for topological evolution, middle life contacts hop from one state to the other, creating narrow trajectories in the topological space. It is interesting to point out how this narrowing of the topological evolution pattern for middle lifetime contacts is not as evident in the synthetic polyQ model. This indicates there is some AR‐specific coding within its secondary structures that leads to a well‐defined configurational change within the middle lifetime. This is contrary to the view of the NTD as having a completely random conformation. The three runs show a clear pattern towards higher fractions of cross relation, indicating an evolution towards higher degrees of entanglement. The topology of long‐life contacts is shown in Figure S14. As the scale τ > 2 μs is close to the duration of the simulation, this scale is subject to heterogeneous behavior caused by kinetic traps, which manifest in the topological space as globules similar to those we have encountered for short lifetime. The same analysis carried out on CR (Figure S15) reveals a similar topological evolution, although the topological make‐up of the two regions is quite different. While NR and CR have similar cross fractions, NR consistently scores higher in parallel, and the CR in series fractions.

In the context of lifetime analysis, one can consider following the evolution of a specific set of residues, and selectively visualize their contact formation dynamics. For example, cysteine residues play an important role in the folding and conformational dynamics of the proteins by forming disulfide bonds and covalently connecting two distinct parts of the chain.^57^ There are 11 cysteine residues in AR‐NTD, which make up for a total of 55 possible cys‐cys contacts. Interestingly, only five of such possible contacts actually occur across our simulation runs. These contacts are by nature very dynamic and often form and break multiple times during a run; however, it is possible to calculate the cumulative time a certain contact is detected across frames in the simulation, in order to see which configurations are most likely to occur. Among the five outstanding contacts, we found contacts ^239^Cys‐Cys^266^, ^239^Cys‐Cys^326^ in which Cys239 stand out as a main contributor. This agrees with previous finding that Cys239 plays an important role in oxidative hemostasis of the AR and aggregation behaviour of the chain.^58^

One might also consider including spatial information encoded in sequence separation, to shed a light on these topological differences. For this purpose, we calculate the average range of contacts for each residue. The distribution *P*(d) of such spatial range (Figure S16 a) peaks at about 50 residue–residue distances, for both regions and all MD runs, indicating once again the high dynamicity of this domain. The most probable range of interaction in NTD is already larger than what is commonly identified as interaction range threshold defined for long‐range contacts in proteins (typically 24 residues). Thus, there is a need for defining a relevant threshold for contact range. Based on these distributions, we identified a threshold for long‐range contacts ($\lambda \geq <d>+\sigma_{d}$ ~ 82 residues). The NR region consistently shows a higher density of such long‐range contacts (Figure S16 b). This might explain the different topological profiles observed for the two regions: short range contacts tend to be in series with each other, as the contact sites are too close together to form entangled loops. Moreover, NR long‐range contacts do not display series fraction at all (Figure S16 c), which might indicate the propensity of the region to consistently interact with itself along its full length (i.e., without creating self‐interacting sub‐domains). Furthermore, middle life long‐range contacts do not consistently show the trajectories in the topological space, which we have identified in Figure 2d. This excludes long‐range contacts as main drivers for conformational change, which therefore happens for smaller spatial scales.

Finally, we note that topological arrangements of contacts have implications for overall shape of the NTD domain. We sampled P‐rich, S‐rich, and X‐rich topologies from topology space of middle‐life contacts from last 500 ns of each simulation. Aligning the structures, we found highly dynamic region composed of residues 50–140 with an obvious structural shift in X‐rich conformations (Figure S17). This also was approved by our RMSD analysis which showed a larger deviation for X‐rich conformations in comparison to P‐ and S‐rich ones. The most obvious finding to emerge from the analysis is that high content of X configuration had a key role in this large structural deviation. In the following section, we take a complementary approach and first identify the NTD shape prototypes and will subsequently study their differences in topology and function.

### 3.4 AR‐NTD adopts several prototypic shapes capable of targeting AR‐LBD

**FIGURE 3:**
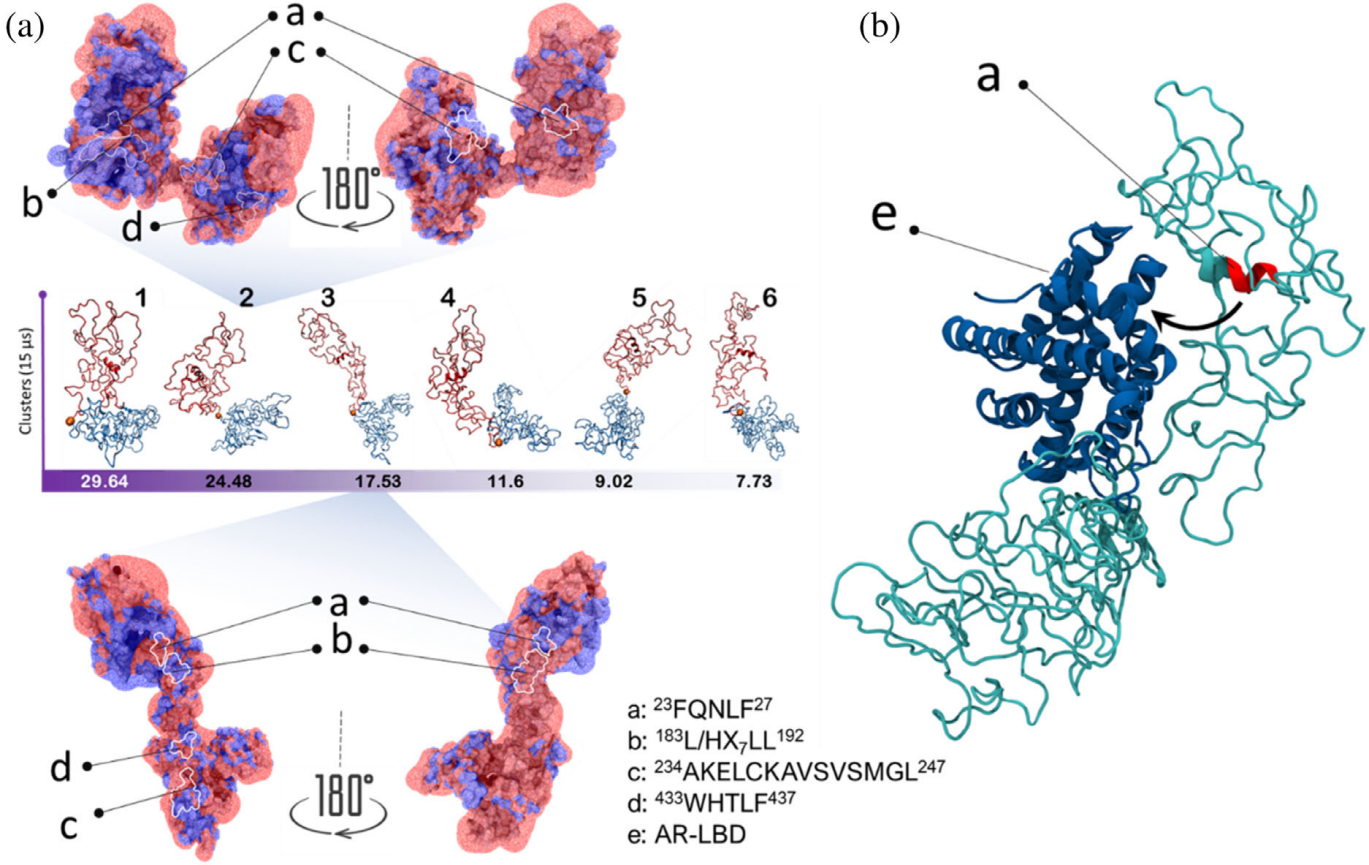
(a) Structures 1–6 are representative AR‐NTD conformations identified by MD simulation and subsequent conformational sampling and clustering of the three trajectories which led to six distinct clusters. Note that the NR‐CR cleft opens and closes as can be best seen in structures 3 and 2, respectively. Surface electrostatic potentials are calculated and mapped as wireframe surface in two colors, red for negative and blue for positive charges. (b) AR‐LBD in complex with representative structure of the cluster 2. LBD docking was performed on all six structures shown in panel a and the highest ranked poses are shown in Figure S20. The identified NTD‐LBD assemblies resemble the recently reported Cryo‐EM images of AR‐NTD^4^ (Figure S21).

Our spatial and temporal analysis study shows that AR‐NTD is dynamic, yet there are regional differences in adopting certain topologies. As a next step, we asked whether these topological transitions contribute globally to the overall shape of the NTD, and whether this would have any impacts on the so‐called N/C interaction which is mediated by ^23^FQNLF^27^ motif and a hydrophobic pocket on the surface of the AR‐LBD. To address this question, we modeled the dynamics of the protein after regional compaction was achieved and sampled the protein conformations. From the sampled conformations, we identified six different classes by performing structural clustering. Figure 3a represents the clusters together with their corresponding occupancy. Looking closer at the circuit topology of these structures, we could categorize them in three groups for whole NTD and two groups for NR region (Figure S18). Structures from the same circuit topology groups were similar in global shape and distinct from other groups. One can readily map the well‐known NTD motifs onto the surface of these structures. The motifs are surrounded by residues that may change during shape transitions (Figure S19). For example, a motif, which is surrounded by positive charges in one NTD conformation, may be surrounded by negative charges in a different shape state. These transitions could be the regulatory mechanism for the binding interactions of these motifs with their partners and be important for their function.

Strikingly, a cleft forms between the CR and NR regions to adopt orientations that resemble opening and closing movements within the AR‐NTD structure (Figure 3a). We performed docking of the identified NTD structures (Figure S20) with LBD (PDB code: 1T7T) and found that the shape of the complex shows striking resemblance to the recently published cryo‐EM image of the full‐length AR.^4^ Particularly, the highest score docking pose from cluster 2 was nearly identical to the reported experimental structure (Figure S21). Interestingly, in the highest score docking pose from cluster 4 (Figure 3b), the ^23^FQNLF^27^ motif is located at a close distance to the AF‐2 pocket, which agrees with the well‐established models of N/C interactions.^1^ Studying best docking poses from all six clusters (a total of 600 poses) reveals that LBD interacts with NTD primarily via the NR region. This interaction dominantly formed either with only NR region or at the cleft. In the latter cases, we do find poses in which the ^433^WHTLF^437^ motif is in close proximity with the AF‐2 site of the LBD. Nonetheless, we do find a small number of clusters where the LBD also interacts with NTD‐CR part only.

### 3.5 AR NTD‐DBD interaction suggests a regulatory role via two distinct binding modes

**FIGURE 4:**
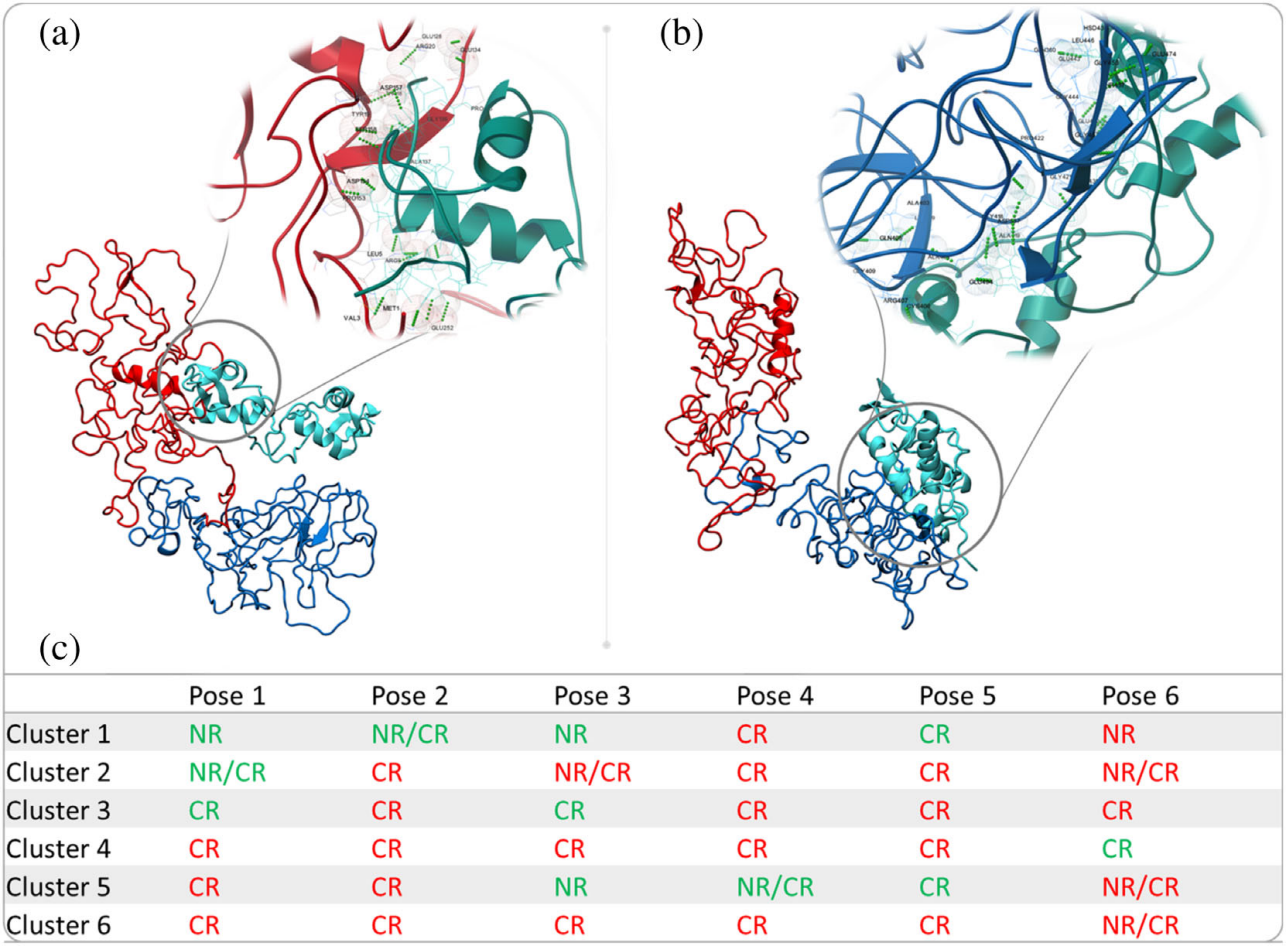
(a), (b) Docking of AR‐DBD to AR‐NTD conformation 1 shown in Figure 4a revealed two binding modes, mediated by NR or CR regions, respectively. In (a) DNA‐binding surface of DBD is exposed and accessible for target DNA while in (b) DNA‐binding surface is fully occupied upon interaction with CR. In detailed analysis of each interacting mode of the DBD with NTD showed that when DBD interacts with NR region both D and P boxes surfaces are accessible. On the other hand, DBD interactions with CR region limited the access to the P box which is responsible for DNA binding.^60^ (c) AR‐DBD docking and interface analysis have been performed for six representative NTD conformations shown in Figure 4a and six highest ranked poses for each conformation are tabulated in (c). When DBD‐NTD interactions involved the DNA‐binding surface of DBD, the interacting NTD regions are colored in red; otherwise, the NTD regions are colored in green.

The AR DNA‐binding domain (DBD) bind to AR response elements, and through this mediates the critical role in the AR transcriptional activity.^59^ We investigated whether and how the identified AR‐NTD conformations could interact with DBD. We used the previously reported crystal structure of the DBD (PDB code: 1R4I) and docked it against the AR‐NTD conformations we have identified, as shown in Figure 4a,b. For each AR‐NTD conformation, highest ranked poses (ranked by cluster size) were selected, and their interaction interfaces were analyzed (Figures 4 and S22). Our data revealed three interaction modes namely (1) interaction via NR, (2) interaction via CR, and (3) interactions that involves the cleft and neighboring NR/CR regions.

Strikingly, CR‐mediated binding typically covers DNA‐binding surface of DBD (residues His553, Tyr554, Ser561, Val564, Arg568, Tyr576, Arg591, Lys592, and Pro595), while NR‐mediated interactions leave this surface exposed. When DBD interacts with NR region both D and P boxes surfaces are accessible. DBD interactions with CR region limited the access to the P box, which is responsible for DNA binding, however, the D box remained exposed and accessible for dimerization. This suggests a model in which CR‐mediated NTD‐DBD interactions could occlude DNA binding, while NR‐mediated interaction would allow for DNA binding. Figure 4c summarizes NTD–DBD interaction modes and their overlap with DNA‐binding site. Our analysis also showed that no cysteine residues were involved in these interactions.

### 3.6 Compact CR binds to an EPI drug

**FIGURE 5:**
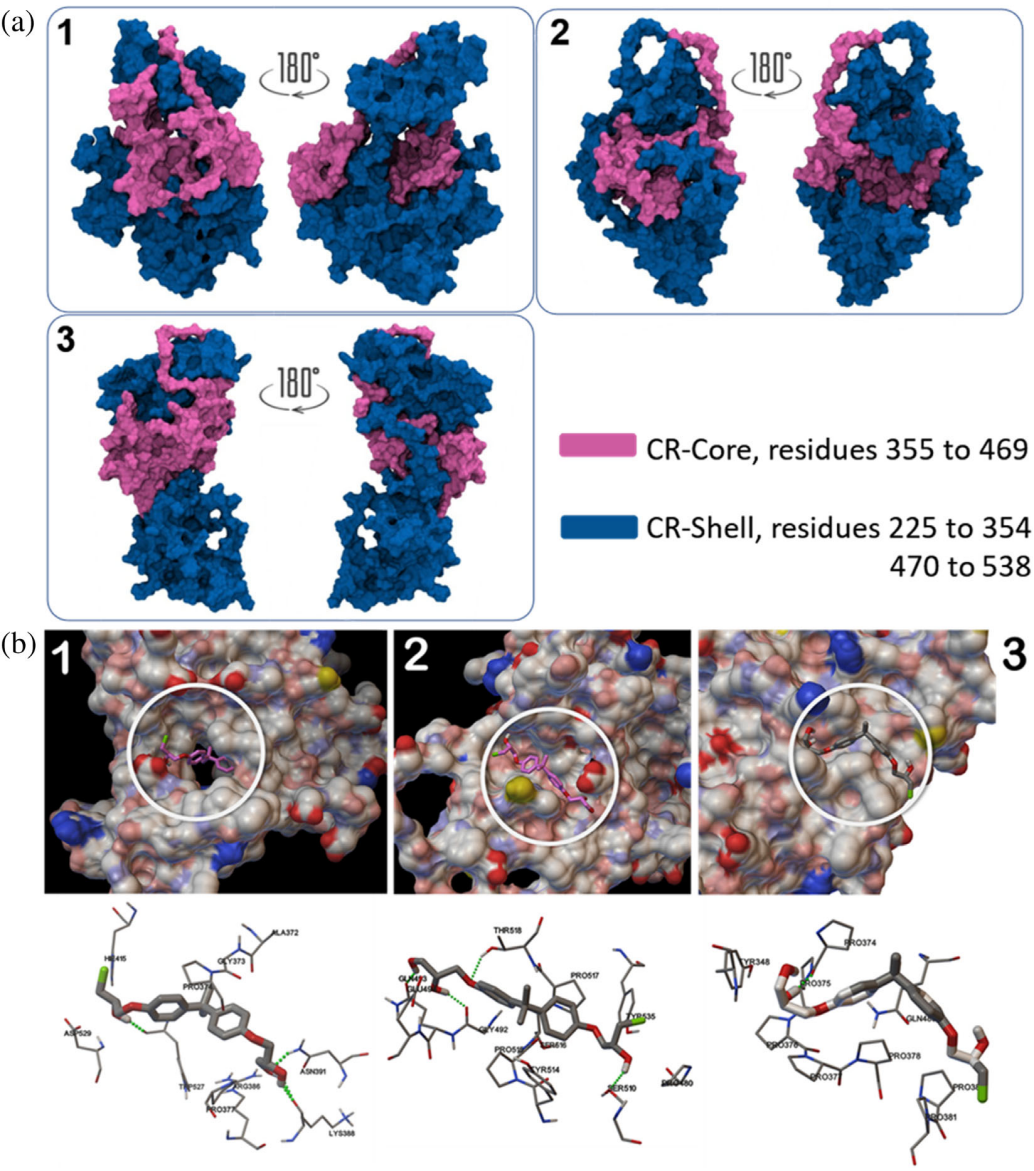
(a) Representative members of three clusters obtained for C‐terminal region (CR). Clustering was done on sampled conformations from the last 3 μs of the simulations, based exclusively on the dynamics of CR region. CR regions are shown in surface representation and CR‐core (residues 355–469) and CR‐Shell (residues 255–354 and 470–538) are colored in pink and blue, respectively. (b) Surface palatability map representations of CR regions from three clusters that show the tri‐dimensional positions of EPI‐001 interacting with distinct regions on the surface of the protein. As it can be seen from the line representations, at least one hydrogen bond is formed between drug molecule and corresponding residues on the surface of the CR. Despite the slightly different interaction sites, drug molecule in all three clusters is interacting with residues that are located within, or adjacent to Tau‐5 functional motif of the N‐terminal transactivation domain.

Due to its medical importance, developing drugs that target AR NTD is an area of intense research; however, the conformational disorder significantly hinders drug development. Only recently, EPI‐001 has been developed as the first inhibitor of the AR‐NTD,^61^ yet we do not know how the drug molecule interacts with the receptor. Interestingly, biochemical studies revealed that the binding happens in the CR part of the AR‐NTD.^52^ To study the drug binding, we clustered the CR region after the regional compaction (last 3 μs) and identified three representative structures. Note that the clusters found in Figure 3a are the result of clustering of the whole NTD and the NR contributes to the heterogeneity due to its dynamics. It is thus expected that CR clustering would lead to a smaller number of representative structures. Modeling the three CR conformations show how the core is enclosed by the shell and how the dynamic shell can mask or expose the core. We performed docking study on the identified CR structures and the EPI‐001 drug and identified the most probable binding modes (Figure 5). The most probable drug‐CR complex is established by three hydrogen bonds. No π‐π or T‐shaped π interactions were observed between the EPI‐001 and the CR region of the NTD. In this binding mode, more than 90% of the interacting residues are located in the Tau‐5 region, which suggests a high affinity of the drug to this key functional motif, in agreement with previous reports.^62^, ^63^ NMR analysis suggested that residues 354–448 are essential for drug binding,^62^ which coincides with core of CR. This is consistent with our most probable model (interacting with residues 372–415). Furthermore, we see that residues from CR shell are also involved in drug binding (residues 527 and 529). This has not been seen in NMR study as the NTD construct used in those studies (residues 142–448) was shorter than the wild‐type and so the shell interaction could not be probed. Moreover, we studied other likely modes and, interestingly, the third CR clusters also showed binding configurations that engage both the CR core (interacting with residues 374–381) and the CR shell (residues 348 and 450) which has a complete overlap with Tau‐5 that is consistent with the NMR data. However second CR cluster showed interactions only with CR shell (residues 492–535) which involves NTD parts not evaluated in the NMR study (and thus cannot be compared).

As a case study to demonstrate the drug effect, we asked whether our identified drug‐NTD interaction can interfere with AR‐NTD for RAP74 interactions. Previous research has found Tau‐5 to be an important interaction site on the AR‐NTD for RAP74 interactions.^7^ Tau‐5 is found to bind helices 2, 2.5, and 3 on the C‐terminal domain of RAP74 (RAP74‐CTD), which form a groove where AR can bind.^2^, ^7^ Importantly, our docking analysis shows that RAP74‐CTD binds the highest ranked CR structure and the binding site overlaps both with the Tau‐5 and the aforementioned EPI‐001 drug‐binding sites (Figure S23). We see a consistent picture with other CR models as well. The data clearly suggest that the drug binding can interfere with RAP74‐CTD binding, in agreement with previous studies.

## 4 CONCLUSION

Our study shows that the AR‐NTD, despite being a disordered chain, can adopt certain topologies and region‐specific behavior. Its structure can be subdivided into two C‐terminal and N‐terminal sub‐regions (CR and NR). The identified topologies, when mapped against known AR interaction sites are significant in how they affect our understanding of the LBD and DBD, as well as drug‐binding interactions that regulate important AR functions. Our structural data are consistent with data from hydrophobic fluorescence probe binding and size‐exclusion chromatography which first suggested the possibility of native AR‐NTD existing in a collapsed disordered conformation, distinct from extended disordered (random coil) and a stable globular fold.^64^ Here, for the first time, we present structural and topological evidence for those speculative models, while also finding significant differences in dynamic behavior between NR and CR. Our observed overall NTD shape dynamics enables the domain to bind to the LBD domain in a way that agrees with recent Cryo‐EM data of the full‐length AR. Strikingly, our structural data also suggest that AR‐NTD can regulate DNA‐binding enabled through competing CR‐ and NR‐mediated DBD‐binding interactions. This is in agreement with earlier biochemical studies, demonstrating that the AR‐NTD can alter DNA‐binding affinity^65^ and the region immediately N‐terminal to the DBD inhibited DNA binding.^66^ Furthermore, in vitro protein–protein interaction (GST‐pull down, unpublished observation) assays show that the removal of the first 187 amino acids of NTD (which corresponds to NR), increases the interaction of the truncated NTD polypeptide with the DNA‐binding domain, suggesting that NR prevents an inhibitory function of CR on DNA binding. Our data suggest a model for CR in which a dynamic shell may enclose or expose the core and thus regulate its binding to cellular partners. Finally, the modeling analysis allowed for the identification of a previously unknown binding site in the CR core and shell of the AR‐NTD, revealing also the functional motifs to which the EPI‐001 drug binds, and how the interaction interferes with co‐regulatory RAP74 protein binding to NTD.

**FIGURE 6:**
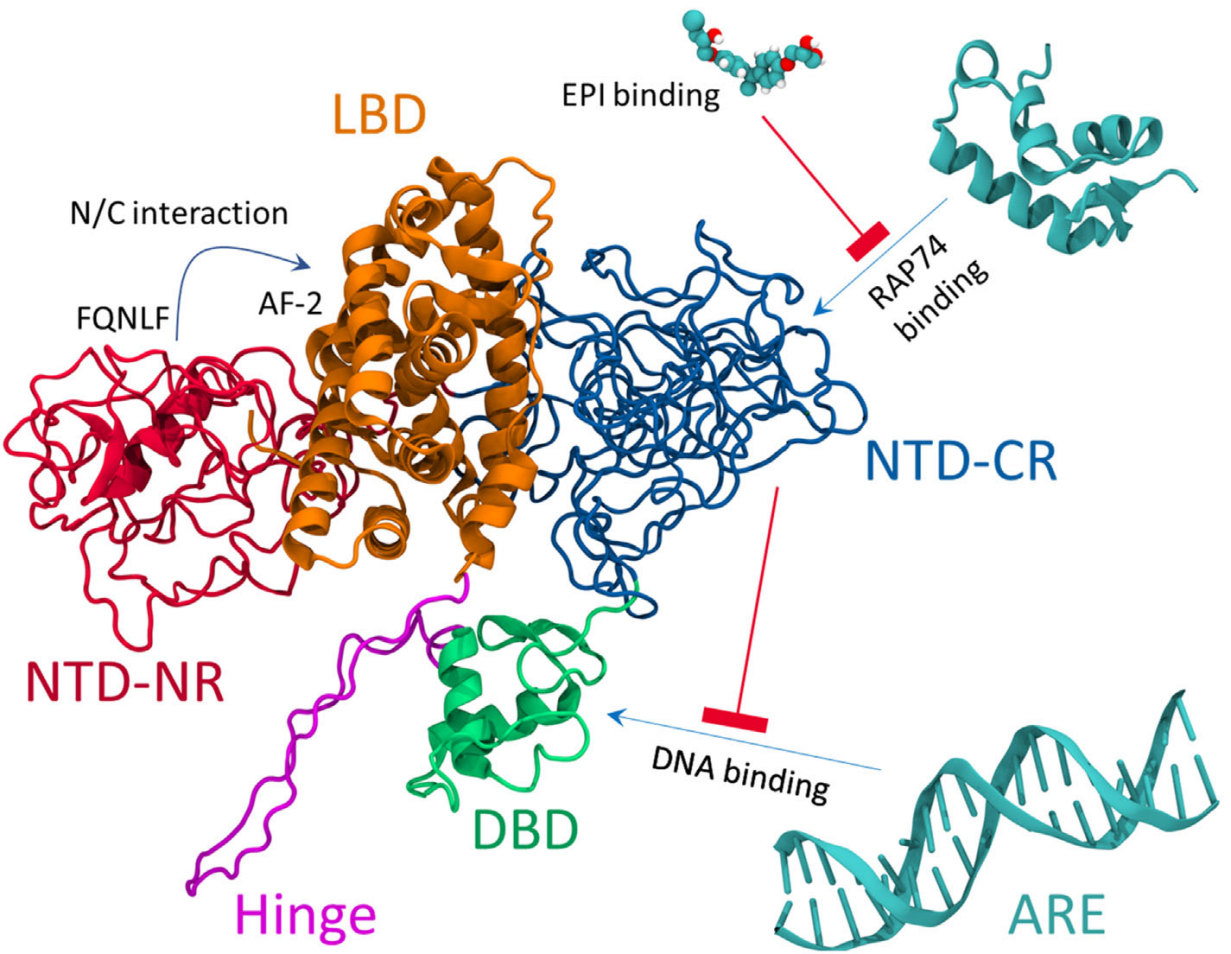
Full length AR structure constructed based on our N‐terminal transactivation domain (NTD) model. In order to construct the full length AR model, full length AR structure was obtained from AlphaFold, the NTD domain (residues 1–538) was cleaved, and the rest of the AlphaFold model was aligned to our NTD–LBD complex. Connections later were made using Chimera software. Our full‐length model shows the expected close proximity between the FQNLF motif on NTD and the AF‐2 pocket of the AR‐LBD which are the main mediators of the formation of a well‐established interaction in AR known as N/C interaction. The spatial localization of the DBD with respect to the CR region is in a way that it allows DBD to move and interact with the CR region. Our model also clearly shows that the hinge region and dimerization interface of the LBD are both accessible for interaction with partner proteins. Finally, we do not see any spatial limitation for the binding of EPI‐001 drug or RAP74‐CTD to the CR region, in line with the proposed mechanism of action of RAP74 and the inhibitory EPI‐001 drug.

Our NTD analysis allowed us to build a model for wild‐type human AR. Figure 6 shows a model constructed using multiple structure alignment approach. Figure 6 also summarizes the key interactions and regulatory mechanisms, suggested by our data. This includes: (1) the well‐established N/C interaction, (2) NTD‐mediated regulation of DNA binding, and (3) drug‐induced inhibition of RAP74 binding to NTD. All these findings are in close agreement with experimental data, as discussed above. The model can open up possibilities for future investigations of AR biology and disease analysis. For example, we speculate that post‐translational modification sites as well as pathogenic mutations^67^, ^68^, ^69^ are typically residing in CR and in close proximity to long life‐time attractive contacts^70^; a hypothesis that can be readily addressed in follow‐up studies. Furthermore, we note that the hinge region is highly exposed in the proposed model. A growing number of evidence has shown that the hinge region plays a crucial role in regulating AR function.^71^ Besides mediating nuclear translocation and DNA binding, the AR hinge region acts as a target for post‐translational modifications^67^ and can recruit some co‐regulatory proteins to affect AR activity.^72^ Structural, biochemical, and cell‐based analysis are required to verify the proposed interactions and mechanistic models, and their relevance to in vivo biology.

Our article sets the stage for future investigations of AR biology, and provides a workflow for analysis of other NHRs. In our current study, we focused on NTD dynamics in isolation. An important future goal would be to study the impact of interdomain interactions, and the role homo/heterodimerization and co‐regulatory protein binding have on the topological dynamics of AR‐NTD.^60^ Highly regulated and complex interdomain interactions in AR and other NHRs could be an example of how cross‐talking between structured domains and disordered regions can regulate complicated cellular functions. Despite differences in primary sequence and thus many structural properties, NTDs of NHRs share certain features and thus comparing our data with experimental data from other NHRs might also be informative.^73^, ^74^, ^75^ In agreement to our data, previous experiments suggest that NTD of NHRs are largely disordered. However, importantly intrinsically disordered structure can range from fully unfolded to partially folded ensembles and in the case of the AR and the mineralocorticoid receptor^76^ NTD regions with more stable structure (2, 7 and present study). One can of course apply the proposed computational analysis workflow on these NHRs and investigate topological similarities and differences between various NHR proteins.

We proposed a novel approach, termed dynamic circuit topology (dCT), to study protein dynamics from a topological perspective. Conformational disorder is one of the main challenges in structural biology,^77^ and yet, disordered proteins represent very attractive drug targets.^78^ Previous attempts at topological description mainly focused on the protein native conformation,^79^, ^80^, ^81^, ^82^, ^83^, ^84^ a choice inappropriate in this context due to the highly dynamic nature of AR‐NTD. Dynamic systems require dynamic approaches and here, we have applied a multi time‐scale analysis to highlight the topological evolution of the system, revealing that the most apparent (elongated) trajectories in the conformational space happens between 0.5 and 2 μs, while the vast majority of AR‐NTD contacts are formed and broken with a lifetime shorter than 0.5 μs. A major technical advancement is the representation of the folding process on the topological landscape. Expressing conformational evolutions in terms of S, P, and X allows for clear visualization and quantification of topological evolution. This reveals that topological trajectories are developed by hopping from one configuration to the other. This happens generally in the direction of decreasing series and increasing parallel and (especially) cross fractions. Although both NR and CR display similar qualitative behavior in this respect, quantitatively they are very different, both from a timescale and a topological point of view. While a narrow topological trajectory is clearly visible for CR without the need of dynamic analysis, it is necessary to disentangle the role of short‐lived contacts to be able to visualize a trajectory for NR. Moreover, the differences in topological make up between the two regions allow us to speculate about how formation of sub‐structures with high contact density arise in AR‐NTD. The higher series fraction in CR suggests that transiently connected sub‐structures may be formed, which are mostly independent of each other. The formation of such sub‐structures might be necessary for folding and target binding. On the other hand, the longer range of residue–residue interactions in NR (and the lack of series contacts for long range contacts) is suggestive of very high flexibility of this region, with possible biological implications; it could be functional, for example, for rapid opening and closing movements in the structure. Overall, we envision that the contact analysis methodology used in this study paves the way for more in depth dynamic structural/topological studies on disordered proteins including other NHRs, creating new opportunities for drug development.

## AUTHOR CONTRIBUTIONS

**Vahid Sheikhhassani:** Formal analysis (lead); investigation (lead); writing – original draft (equal); writing – review and editing (equal). **Barbara Scalvini:** Formal analysis (equal); Investigation (equal); writing – original draft (equal); writing – review and editing (equal). **Julian Ng:** Resources (supporting); writing – review and editing (equal). **Laurens Heling:** Investigation (supporting); writing – review and editing (equal). **Yosri Ayache:** Investigation (supporting). **Tom Evers:** Investigation (supporting); writing – review and editing (supporting). **Eva Estébanez‐Perpiñá:** Resources (supporting); writing – review and editing (supporting). **Iain McEwan:** Resources (supporting); writing – review and editing (supporting). **Alireza Mashaghi:** Conceptualization (lead); funding acquisition (lead); investigation (equal); supervision (lead); writing – original draft (equal); writing – review and editing (equal).

## CONFLICT OF INTEREST

The authors declare no competing interests.

## Supporting information


Appendix S1
Additional supplementary information can be found via the following link: https://github.com/TheMashaghiLab/publications
