# Supplementary material for: Topological dynamics of an intrinsically disordered N‐terminal domain of the human androgen receptor
Source: Protein Sci. 2022 May 26;31(6):e4334. doi: 10.1002/pro.4334 (PMC9134807; doi:10.1002/pro.4334)
Supplement: Supplementary file 1 — Appendix S1 Additional supplementary information can be found via the following link: https://github.com/TheMashaghiLab/publications [file PRO-31-e4334-s001.docx]

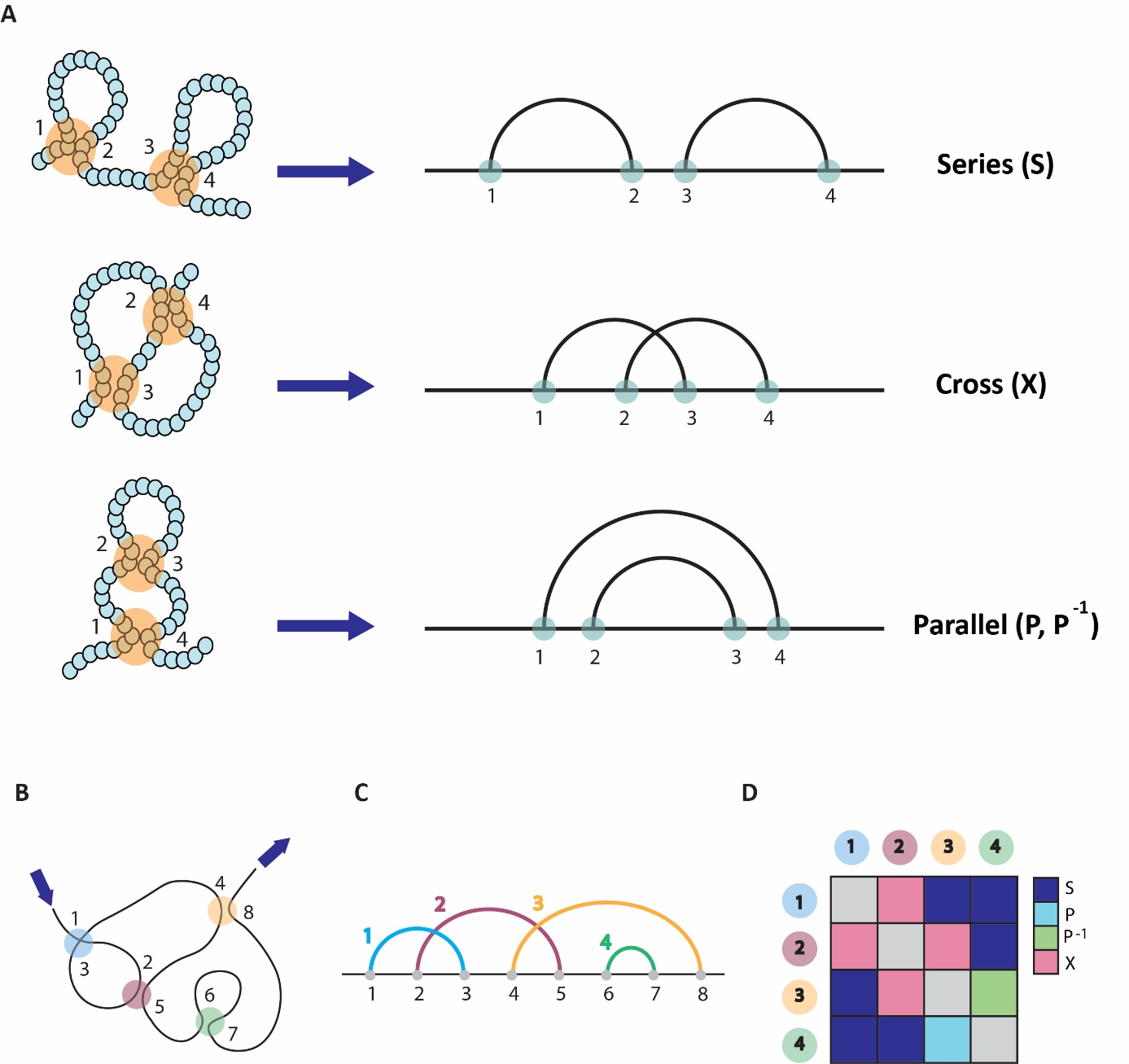


Figure S1. Circuit topology is a contact-based theoretical framework for the characterization of folded chains. (A) Schematic representation of the three topological relations: series (S), parallel (P, P-1) and cross (X). Each pair of contact can participate in either one of these three relations. These relations are symmetric (if contact A is in series relation with B, then B is in series relation with A), with the exception of the parallel relation. For this reason, we specify two possible representation for the parallel relation, parallel (P) and inverse parallel (P -1). The significance of these two relations is intuitive: if contact A is in parallel relation with contact B (contact A is enveloped by contact B), then contact B is in inverse parallel relation with contact A (contact B envelops contact A). Despite this different representation, P and P-1 refer to the same topological arrangement in the chain; therefore, for the sake of the analysis presented in this paper, P and P-1 are considered as belonging to the same topological class. The diagram representation displays all contact sites following the order in which they appear if we scan the chain from left end to right end. Contacts are represented by arches connecting the contact sites. (B) Schematic representation of an hypothetical folded chain. C Linear diagram of the folded chain displayed in B. From the diagram, it is easy to identify the topological relations shown in A. D Topology matrix of the chain displayed in B. The topological matrix stores the topological relations between all possible pairs of contacts in a folded chain. By counting the number of matrix elements which belong to each topological relation, we retrieve what are the percentages of S, P and X contacts within the folded chain.


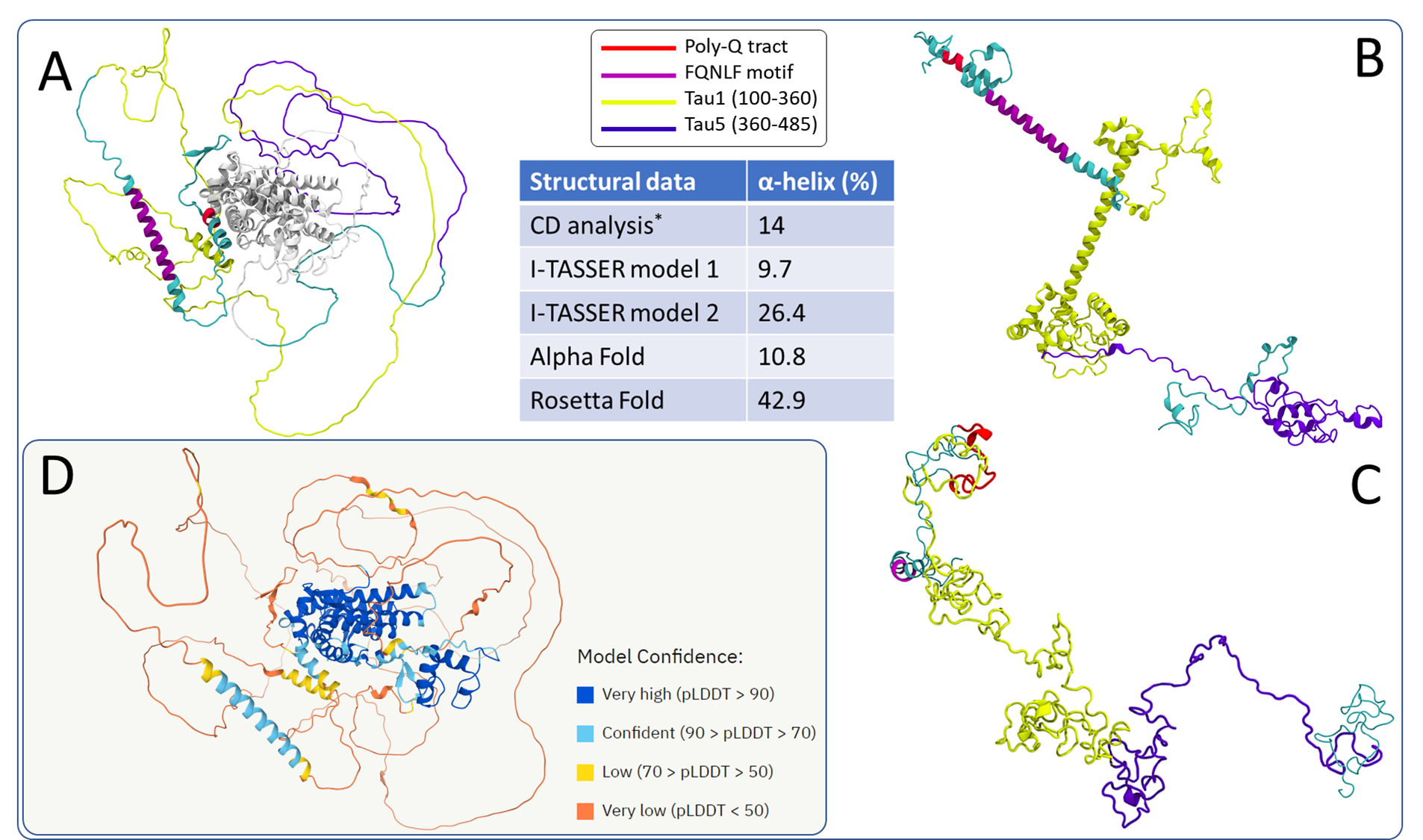


Figure S2. AR-NTD structure predicted using different prediction algorithms. Structures in (A) to (C) show the best ranked models from Alpha fold, RosettaFold and I-TASSER servers respectively. (D) shows the confidence level of the prediction by Alpha fold server. For NTD region (residues 1 to 538), the confidence level is estimated to be less than 50 (colored region in orange), which is verified as very low confidence. Inset table: Comparison between α-helix content of the models predicted by different algorithms with Circular Dichroism data calculated from experimental analysis of the NTD. CD data was obtained from [1]. Among the predicted models, model 1 from I-TASSER and AlphaFold model showed closer values to CD data. Considering very low confidence level of AlphaFold model, model 1 from I-TASSER was chosen for performing MD simulations. The Rosetta Fold generated model does not match the experimental data.


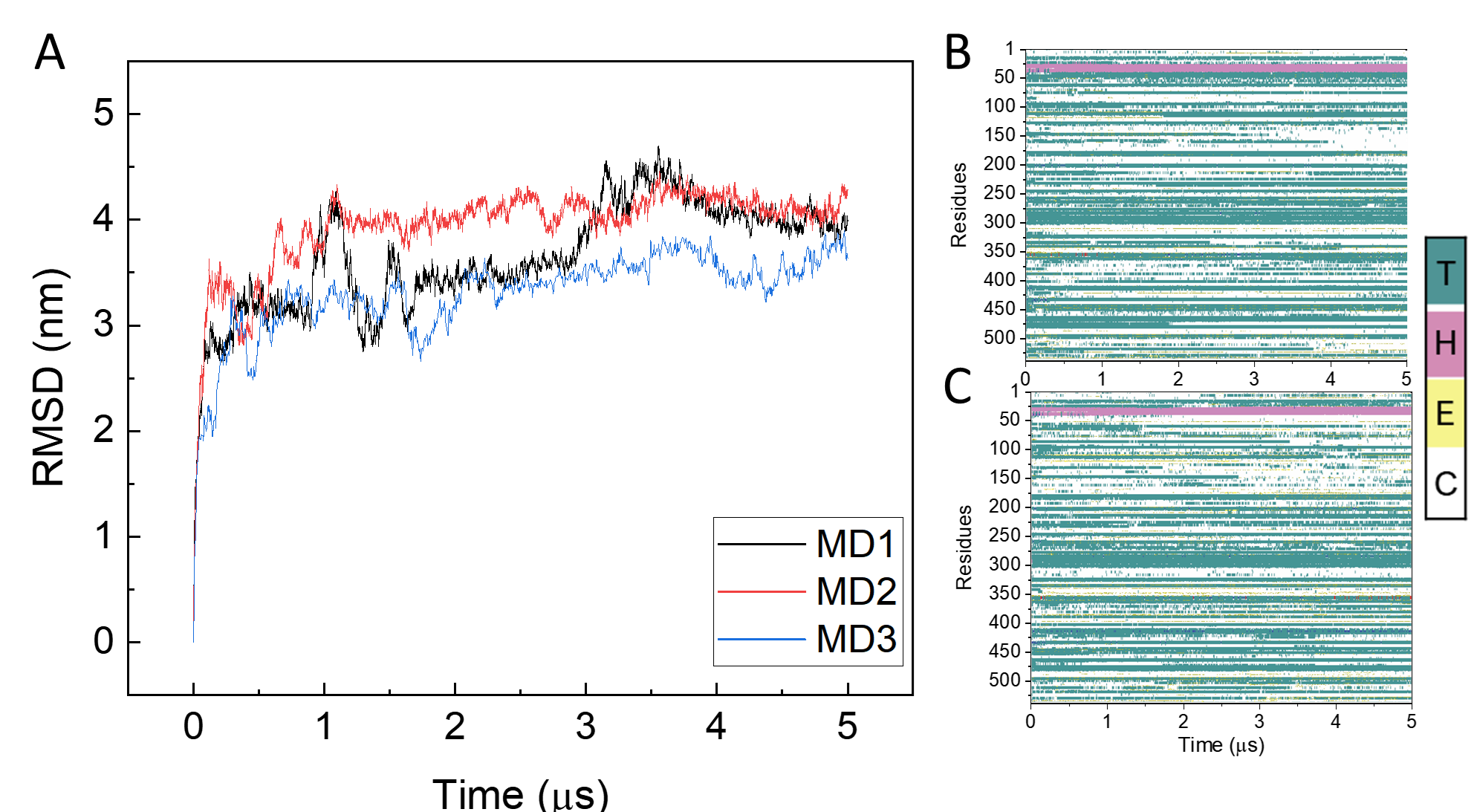


Figure S3. Time evolution of root mean square deviation (RMSD, A) and secondary structure changes. Secondary structure evolution of NTD during 5 us of simulation are shown for MD2 (B) and MD3 (C). As it can be seen from both graphs, turn (T) and random coil (C) secondary structure elements are dominant over the whole period of the simulation.

Figure S4. Average values of solvent accessible surface area calculated per each amino acid. The data is averaged over the last 3 µs of the 3 runs. Vertical lines show the range of amino acids positioned in the dip.


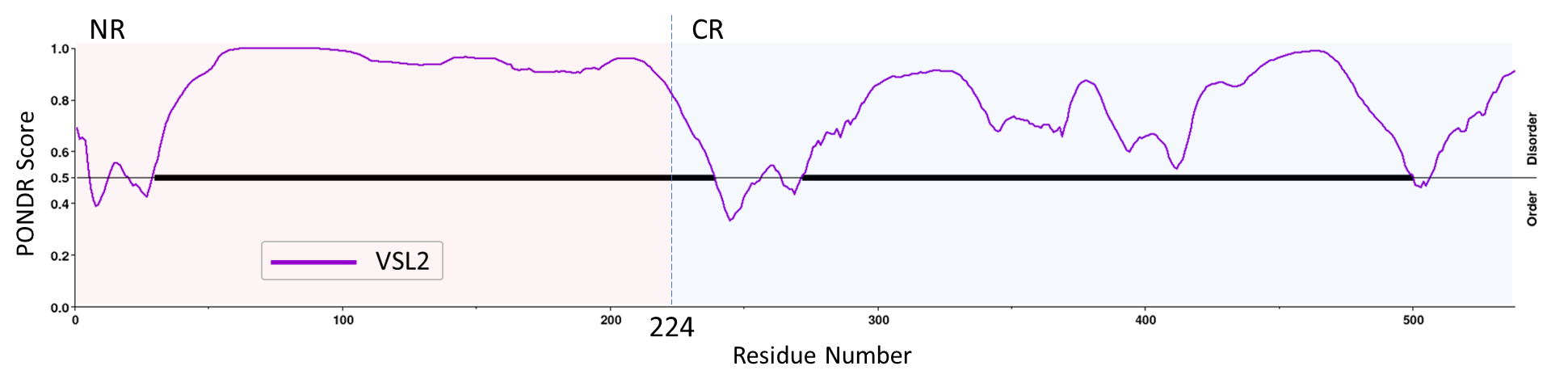


Figure S5. Structural disorder analysis of AR-NTD obtained from PONDR VSL2. Sharp drops in graph highlights potential protein-binding regions.


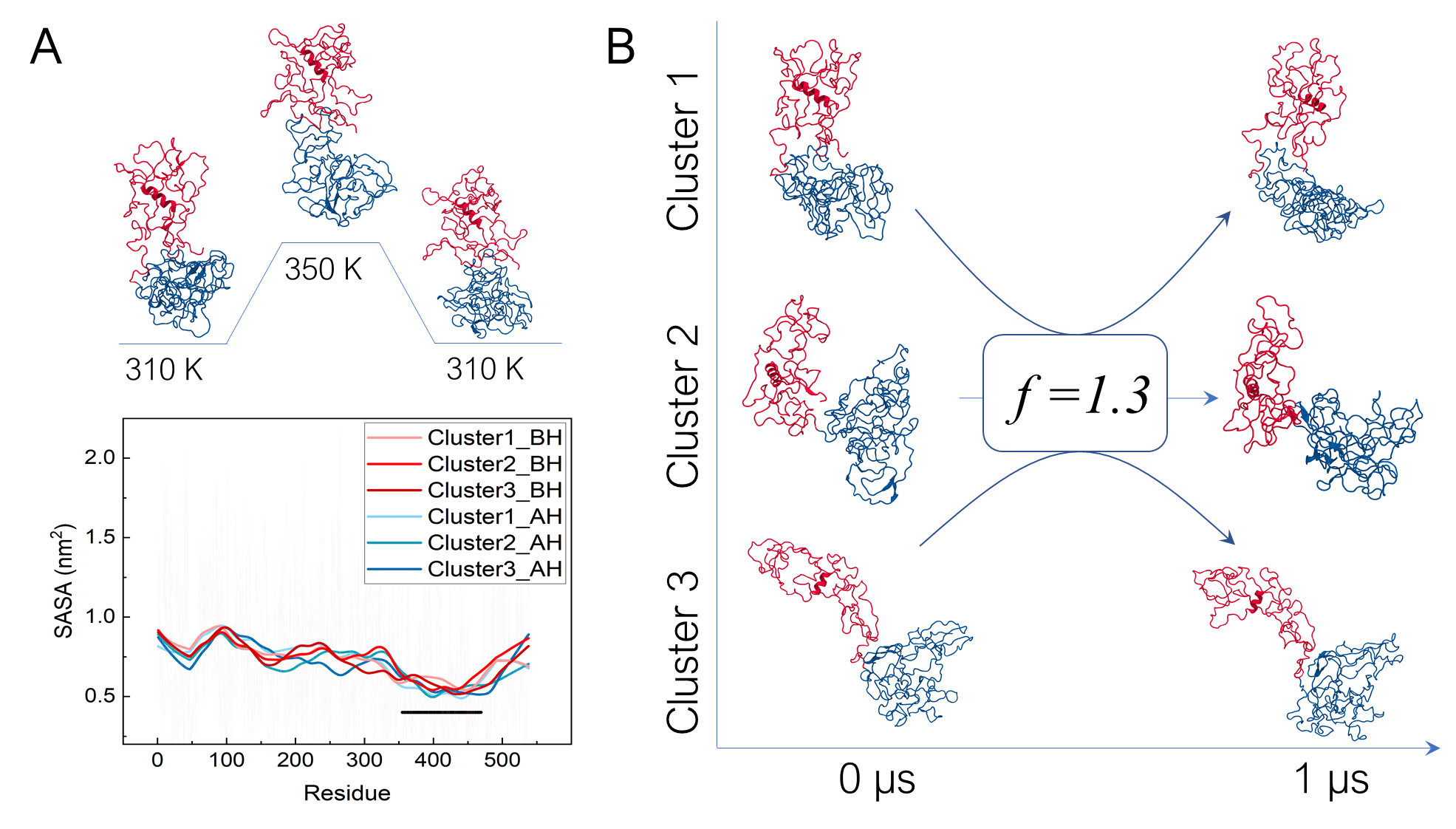


Figure S6. Heat stress and modified f parameter analysis of robustness of two disjoint compact regions formation in AR-NTD. A) To further demonstrate that the formation of disjoint regions does not depend on the choice of initial configurations, we melted the representative members of first 3 clusters using simulated annealing method, equlibiriated at 350 Kelvin, and then ran the simulation for 1 µs after cooling down to physiological temperature. Melting opened up the compact structures specially the CR region. The subsequent simulations however recovered the NR/CR and compactness of the CR in all three experiments. SASA analysis also showed a nice match for before heating (BH at 310 K) and after heating (AH at 310 K) conditions. B) After performing MD simulation with modified f parameter, we found that our observation of NTD folding to two disjoint regions does not depend on the f parameter of the force field, which encodes the interactions between the polypeptide and the surrounding solvent.


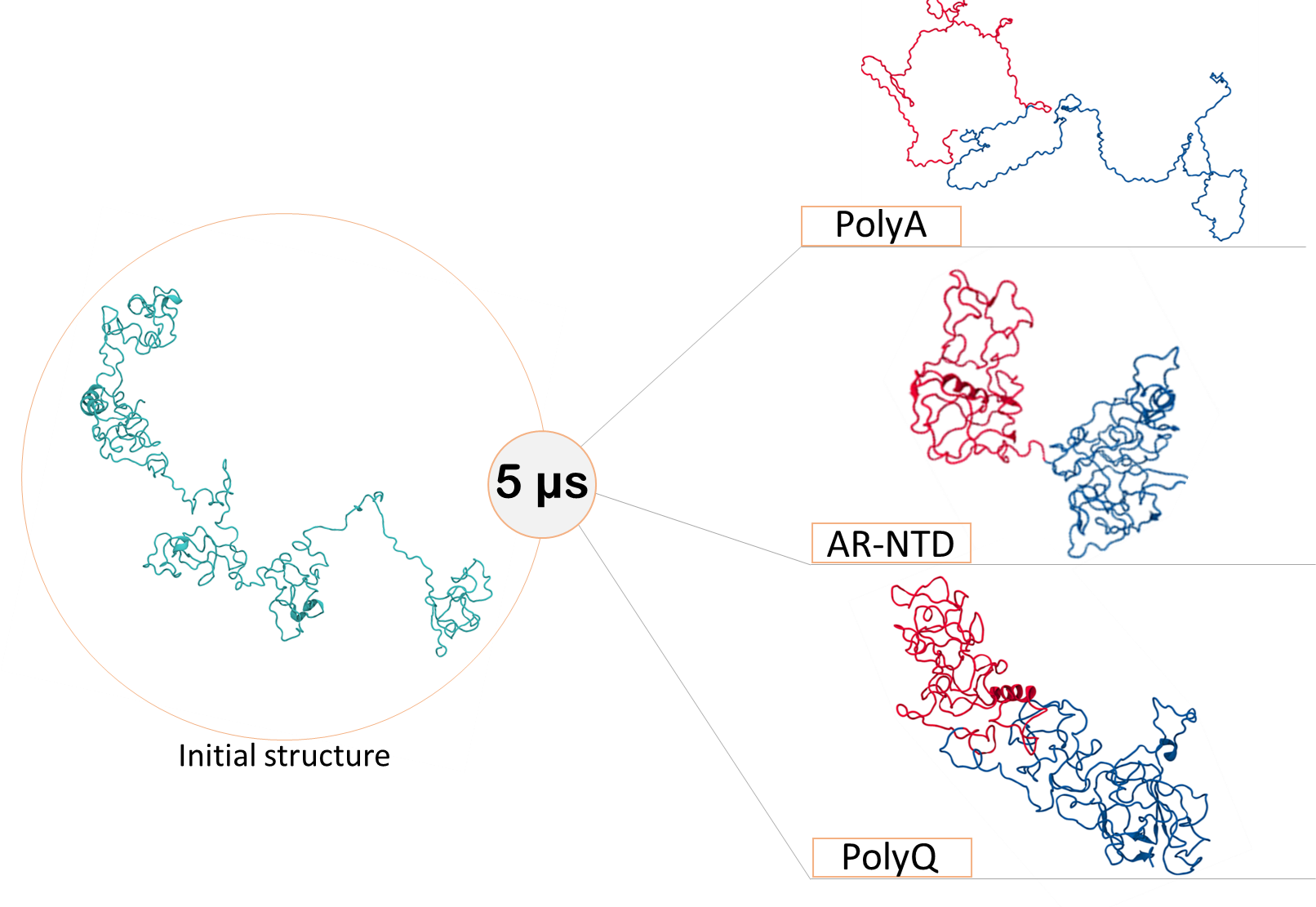


Figure S7. Cartoon representation of the structural evolution of AR-NTD compared to the structure of polyA model of AR-NTD and polyQ model of AR-NTD chain after 5 µs of the simulation. The models were made by taking the precited initial structure of AR-NTD and mutating all to polyA or polyQ residues, respectively.


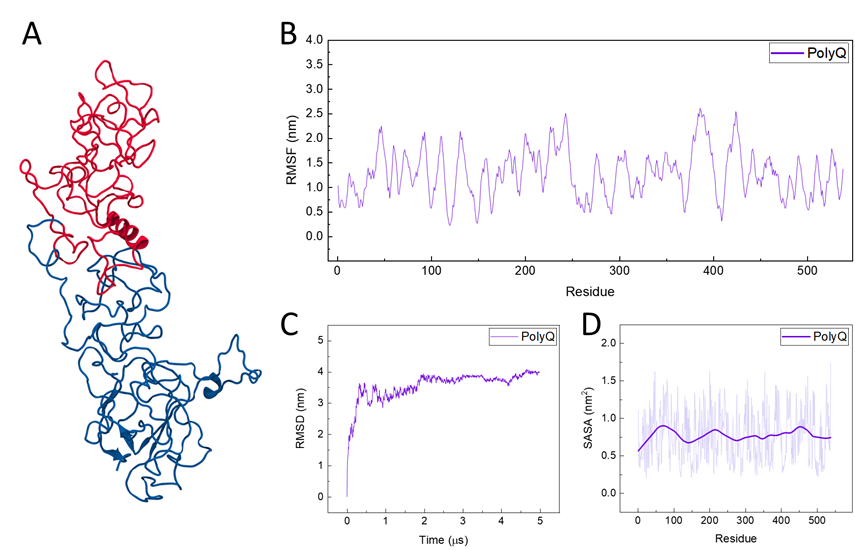


Figure S8. (A) representative member of largest cluster of polyQ chain which is colored in red and blue based on the number of residue numbers of NR and CR regions respectively. (B) root mean square fluctuations analysis of polyQ chain per residue, (C) time evolution of the root mean square deviation of the chain and (D) average solvent accessible surface area per residue calculated from the last 3 µs of the simulation.


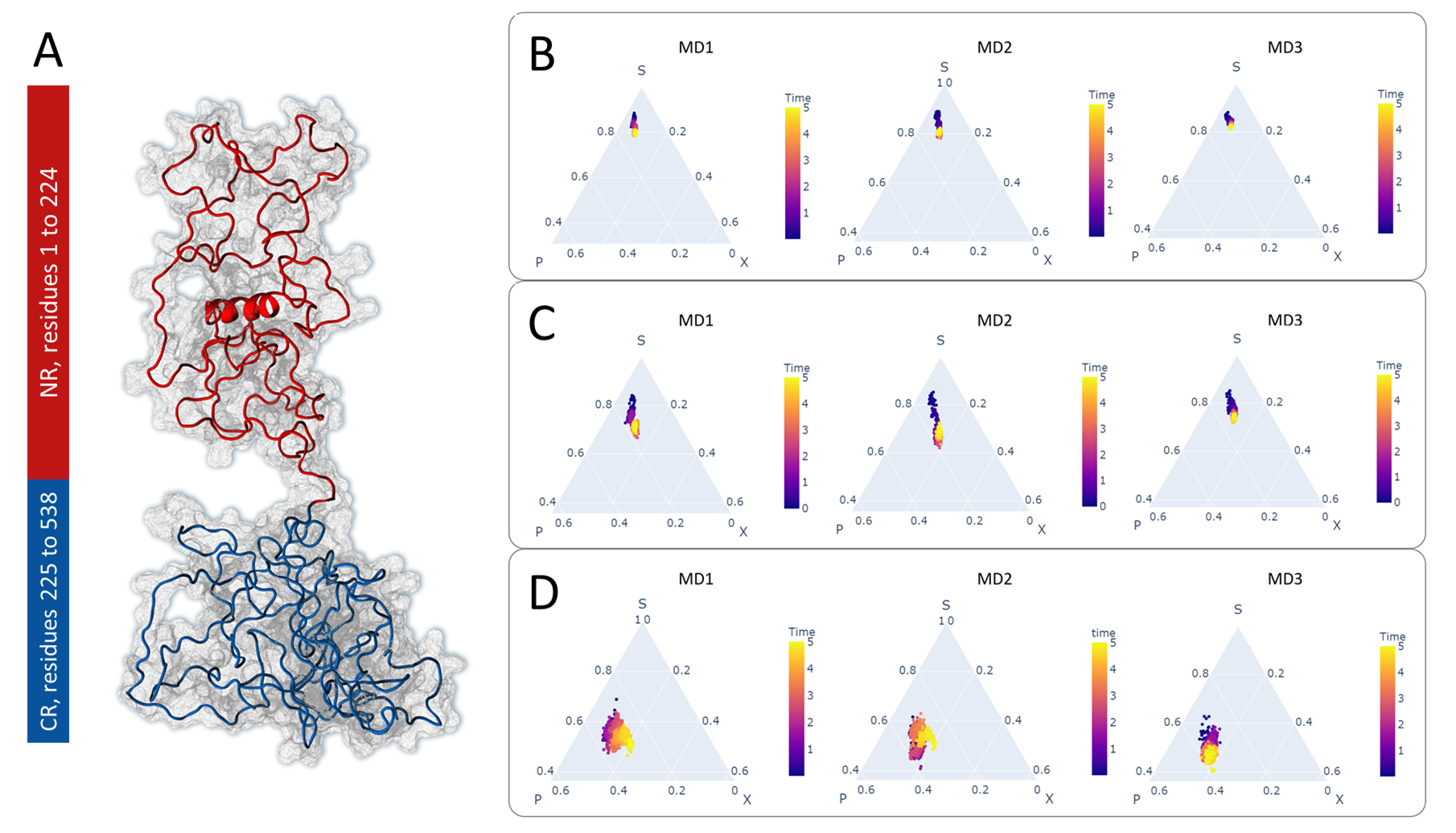


Figure S9. (A) cartoon representation of NR (residues 1 to 224) and CR (residues 225 to 538) colored in red and blue respectively. (B-D) Ternary plot representation of the P, S and X changes. (B) shows these changes for whole structure, (C) for CR region and (D) for NR region. Color bar represents the time duration of the MD simulations.


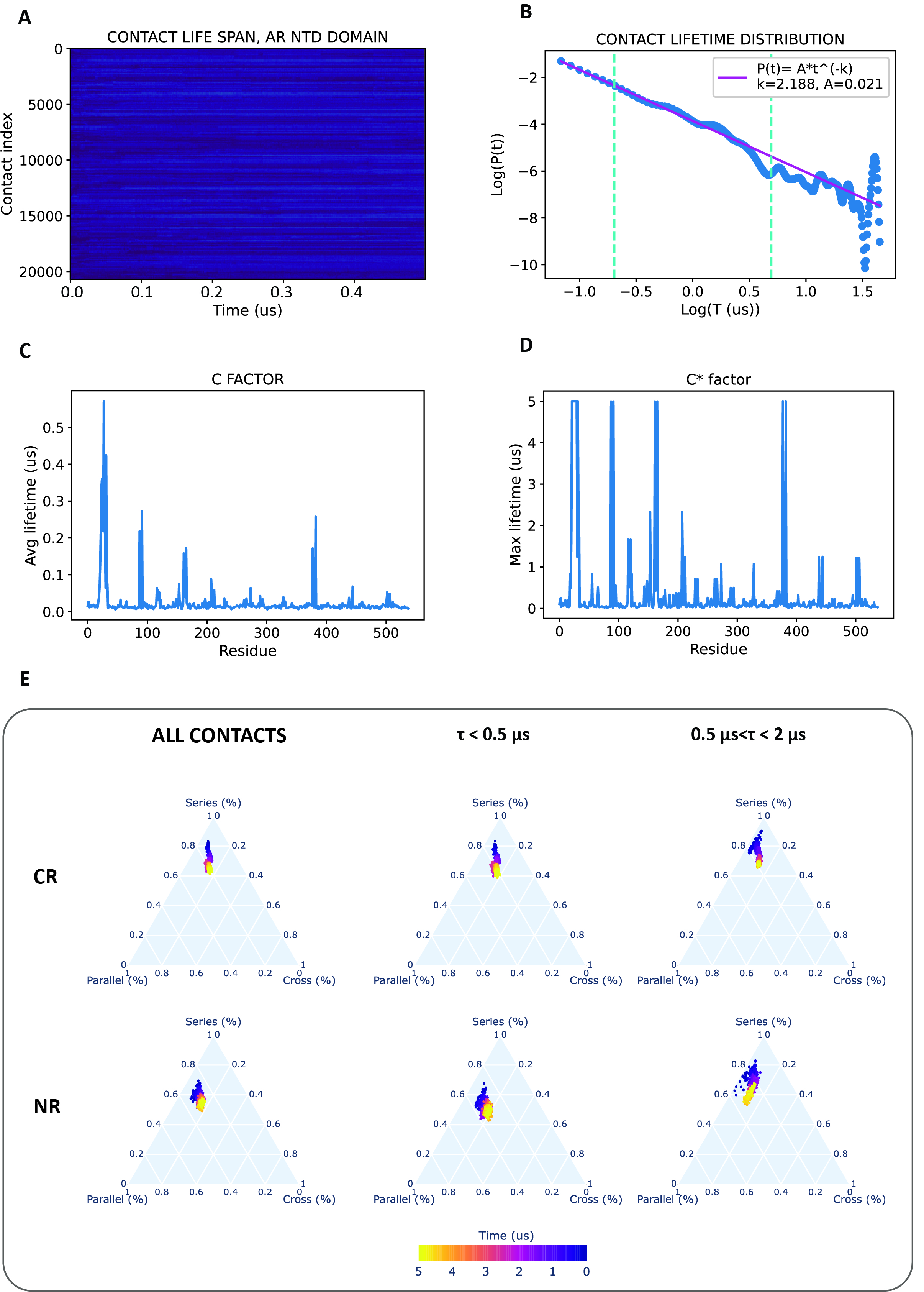


Figure S10. Dynamic CT analysis for the synthetic polyQ model. (A) Kymographs of all contacts formed during the simulation, in NR and CR regions. (B) Log-log plot of contact lifetime distribution, fit to scale-free power law. (C) C factor of contact life-time experienced by each residue. (D) C* factor of contact life-time experienced by each residue. (E) Triangular plot of all contacts within NR and CR in the S/P/X space for short (τ < 0.5 µs) and middle (0.5 µs < τ <2.0 µs) lifetime contacts.


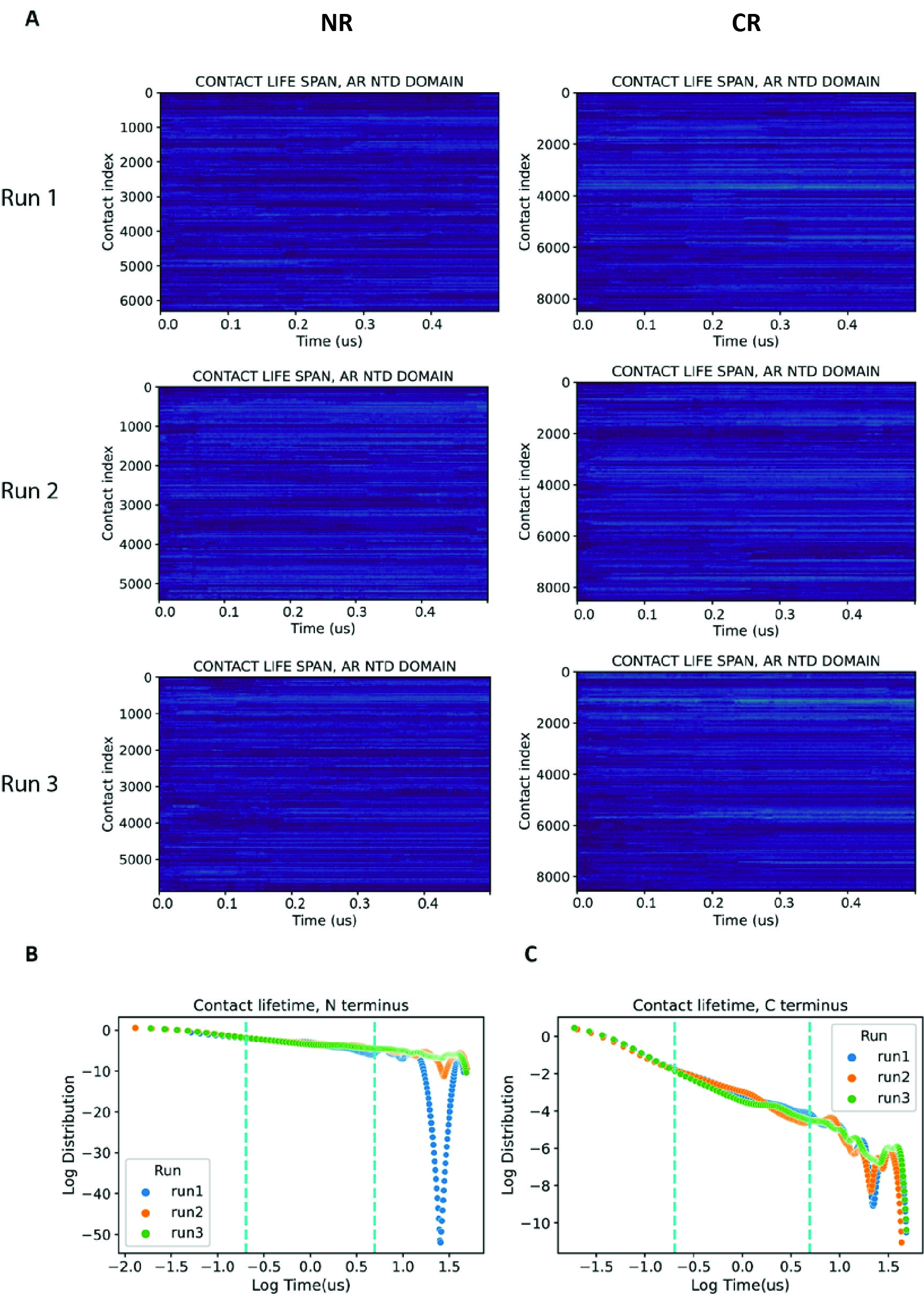


Figure S11. Contact lifetime distributions for NR and CR, AR NTD. (A) Kymographs of all contacts formed during the simulation, in NR and CR regions, for all three runs. (B) Log-log plot of NR contact lifetime distribution from these runs. (C) Log-log plot of CR contact lifetime distribution of these runs.

**
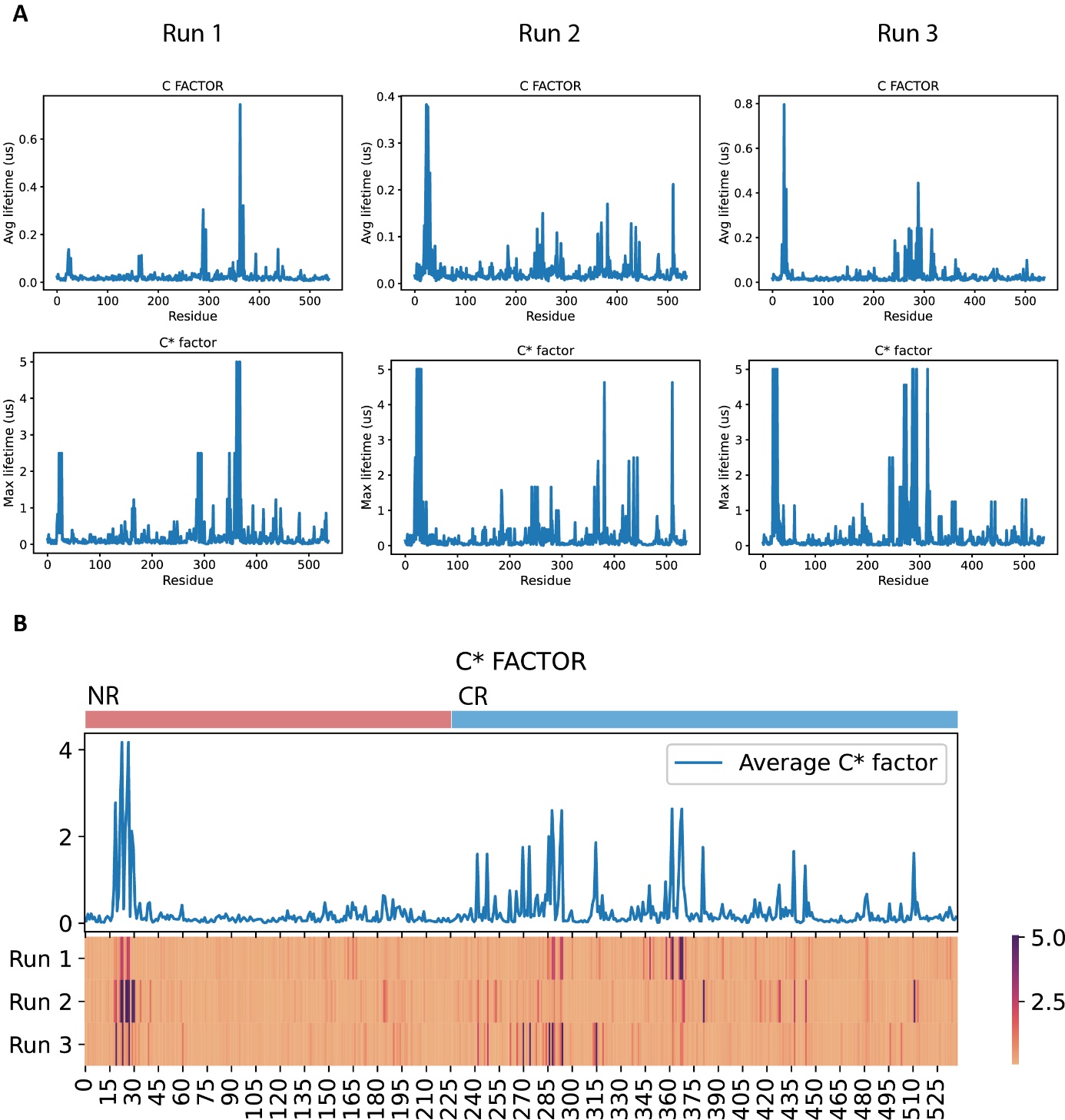
**

Figure S12. C and C* factors. (A) C factor and C* factor (maximum contact lifetime experienced by each residue) for all three runs. (B) Heatmap of C*factor for all runs, with plot of C* factor averaged over all three runs.


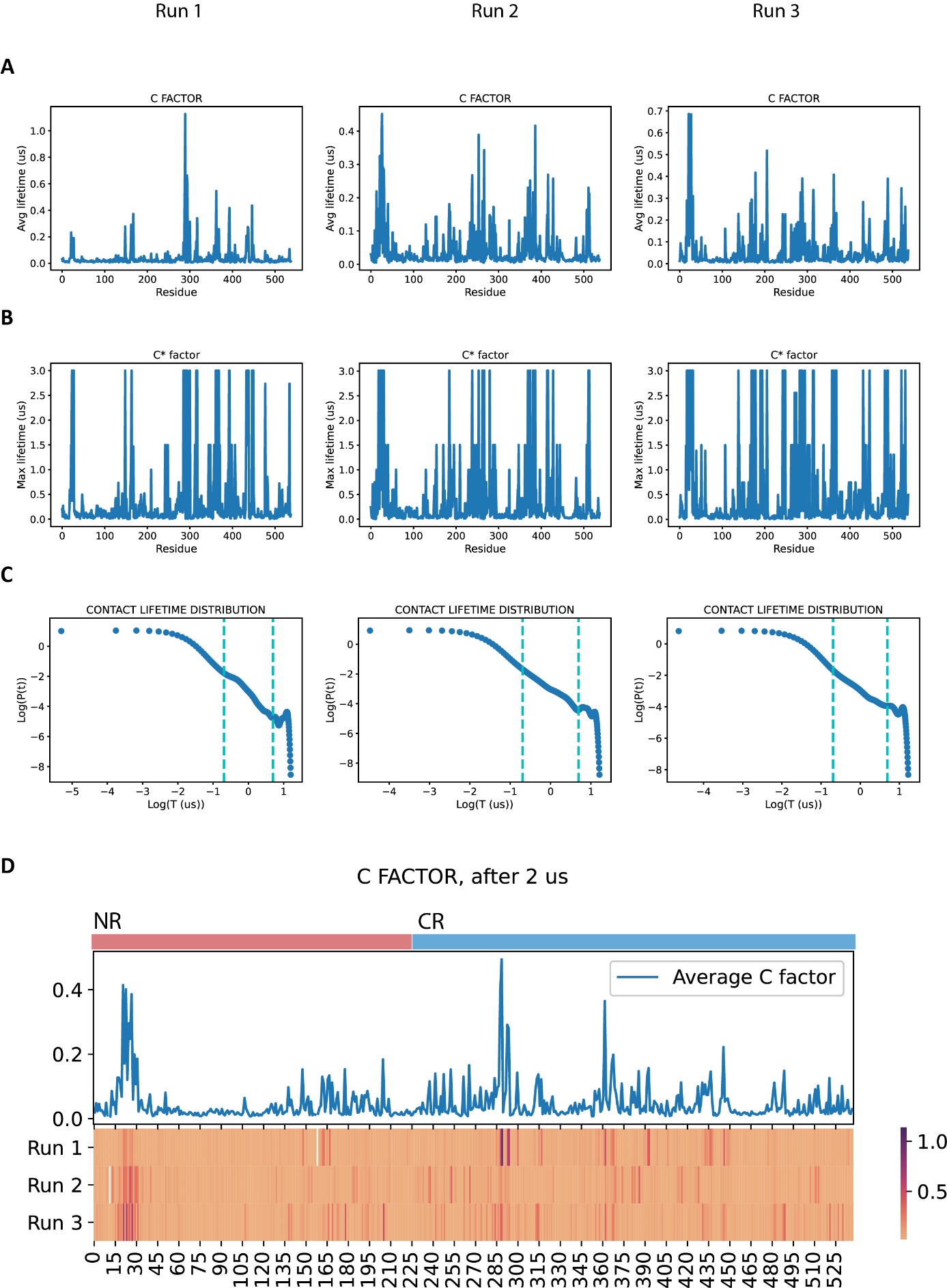


Figure S13. Contact lifetime analysis after 2 µs. (A) C factor for all three runs, for MD frames ranging from 2 to 5 µs. (B) C* factor for all three runs, for MD frames ranging from 2 to 5 µs. (C) Log-log plot of contact lifetime distribution, for MD frames ranging from 2 to 5 µs and for all three runs. (D) Heatmap of C factor for all runs, with plot of C factor averaged over all three runs (for MD frames ranging from 2 to 5 µs).


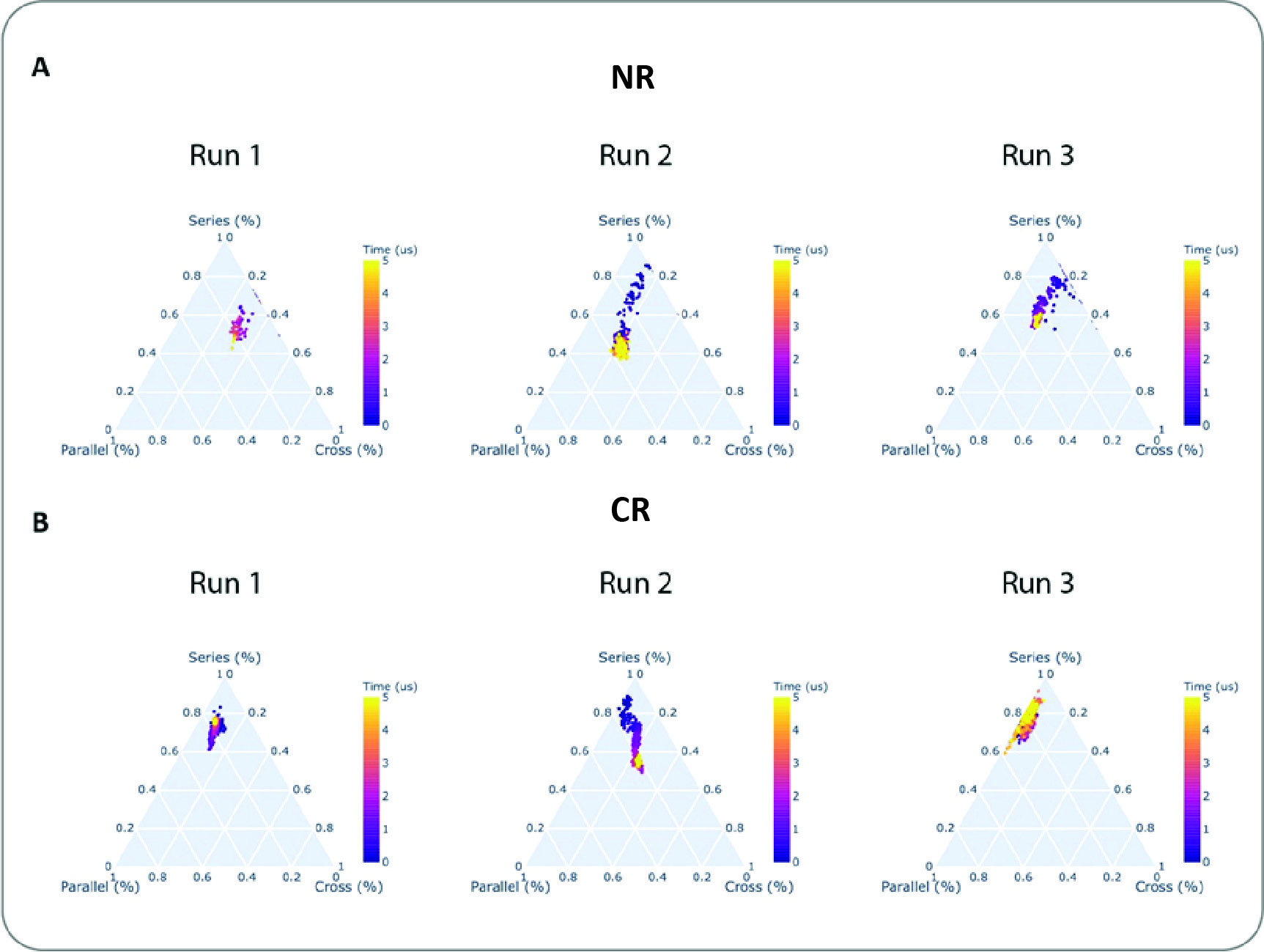


Figure S14. Long lifetime (τ > 2 µs) topological evolution. (A) Triangular plots of NR S/P/X plot for long life contacts (τ > 2 µs). (B) Triangular plots of CR S/P/X plot for long life contacts (τ > 2 µs).


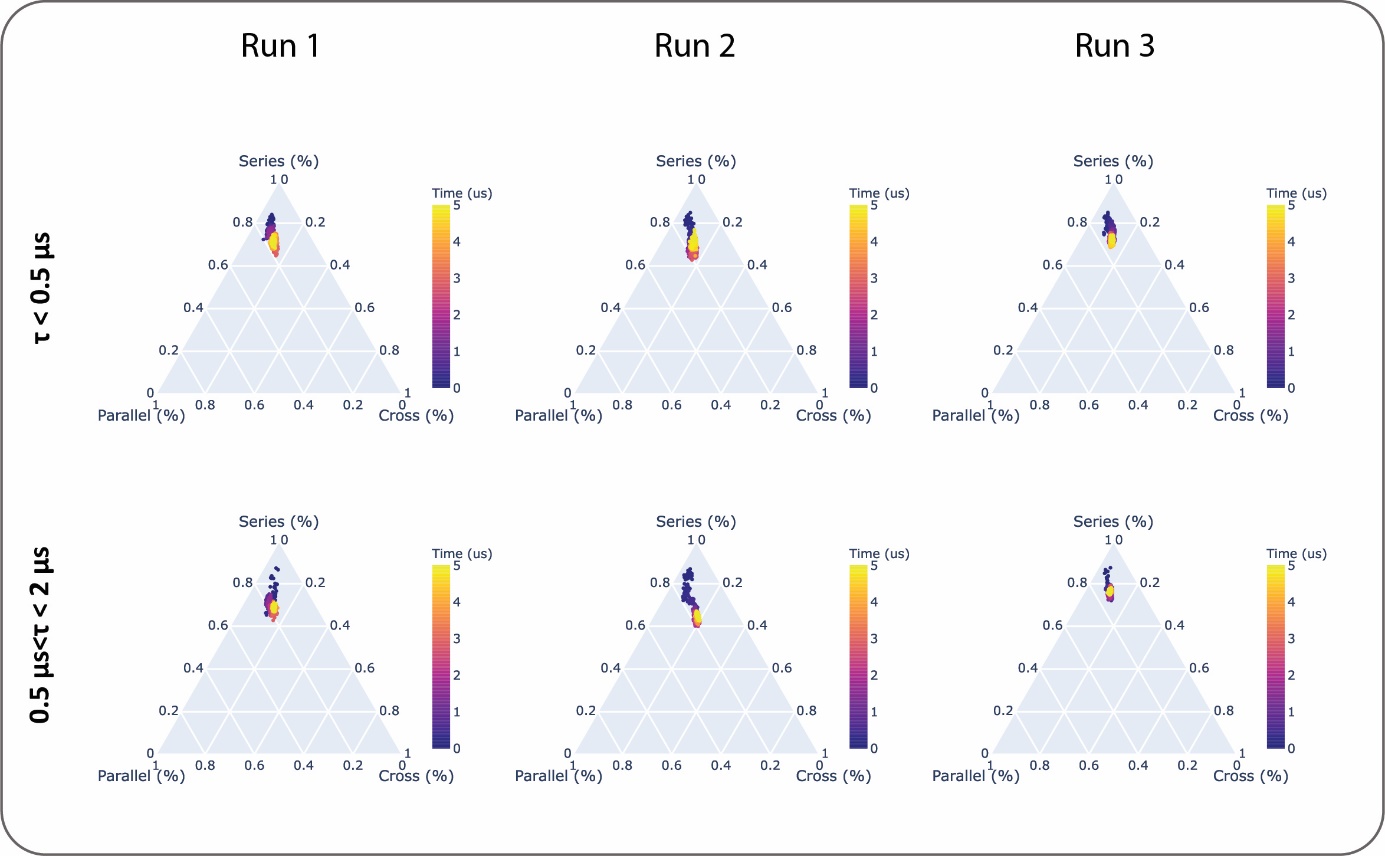


Figure S15. Topological evolution of CR by time regime. Triangular plot of the CR S/P/X space for short (τ < 0.5 µs) and middle (0.5 µs < τ <2.0 µs) lifetime contacts.


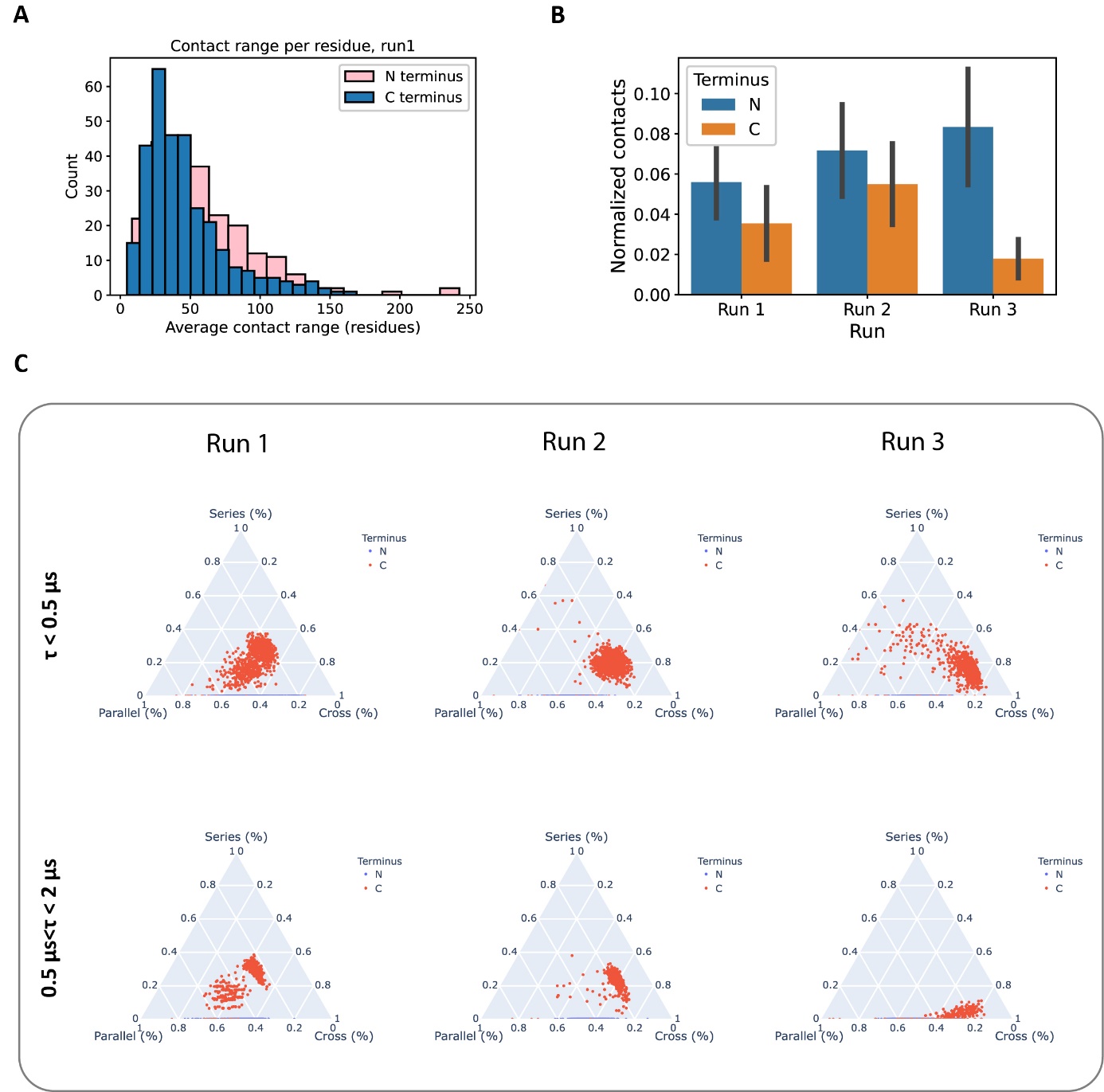


Figure S16. Topological analysis of long-range contacts (~ 82 residues) for NR and CR regions. (A) Distribution of average contact spatial range by residue, for NR and CR, run 1. (B) Bar plot of normalized number of long-range contacts (λ > 82 residues), for NR and CR, all runs. (C) Triangular plot of the NR and CR S/P/X space for short (τ < 0.5 µs) and middle (0.5 µs < τ <2.0 µs) lifetime long range contacts. Series fractions are equal to zero for long range NR contacts, therefore the NR topological evolution degenerates to a line in the topological space.


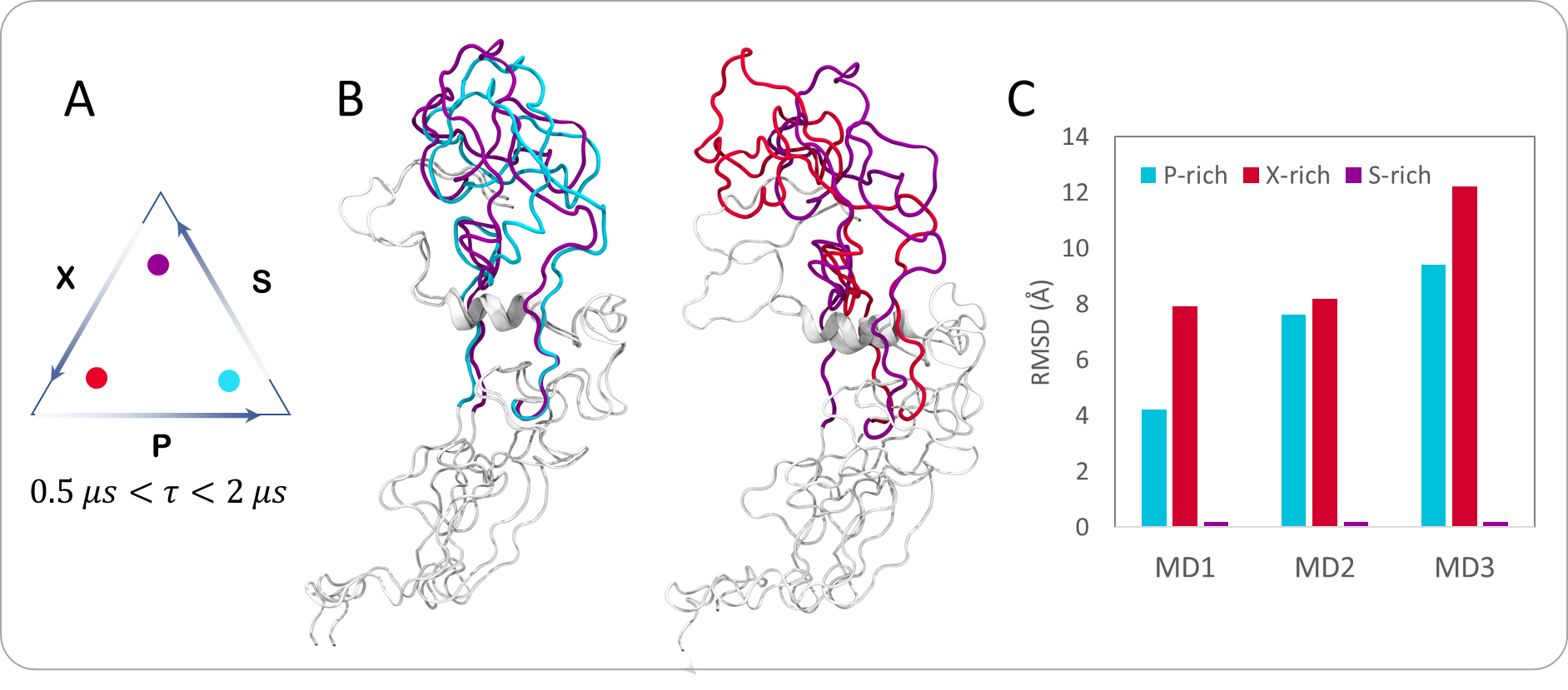


Figure S17. (A) schematic representation of sampling point in topology space for P, S and X-rich conformations which are colored in cyan, purple and red respectively. We sampled structures from topology space of middle-life contacts from last 500 ns of each simulation. Looking at the ternary plots, for each P-rich, S-rich and X-rich topologies we extracted one corresponding structure from the MD trajectory. Structures were superimposed and difference between them were observed visually and also quantified using RMSD (selecting S-rich structure as a reference frame) calculation, (B) cartoon representation of corresponding P and X-rich structure superimposed on S-rich conformation as a reference structure, (C) RMSD analysis of three structures for each simulation showed a large deviation for X-rich conformation.


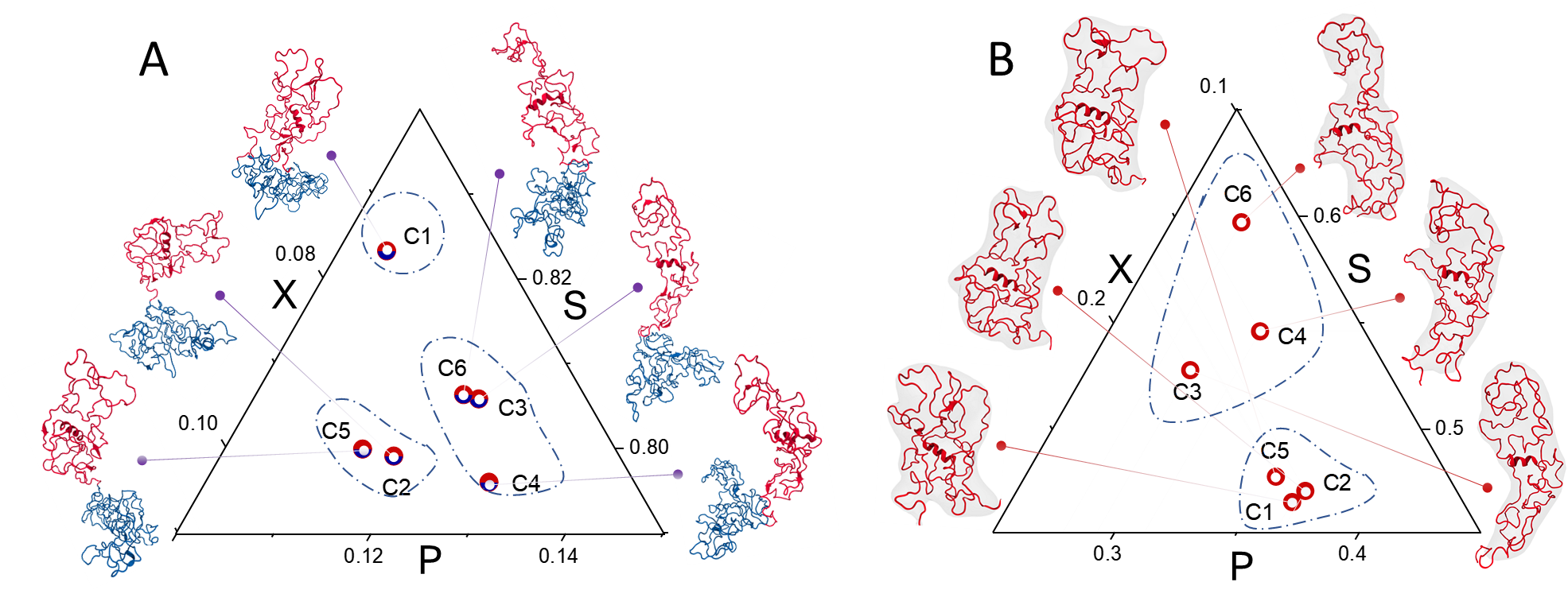


Figure S18. Ternary plots representing the P, S and X content of six selected structures from the MD trajectory calculated for (A) whole AR-NTD and (B) NR region. Corresponding structure with each data point is linked with solid lines.


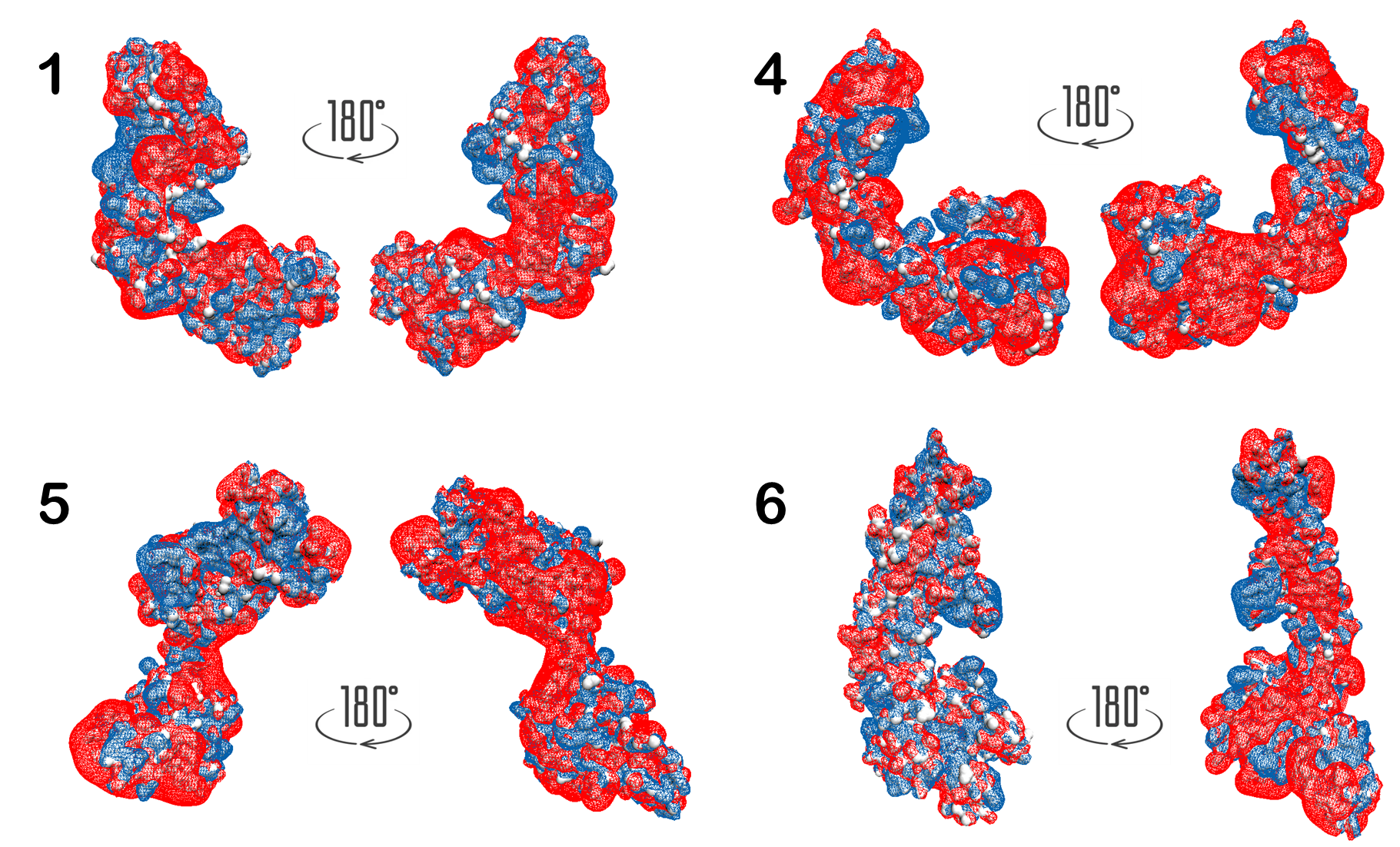


Figure S19. Wireframe surface representation of the surface electrostatic potential. Negative potential is colored in red and positive potential is colored in blue. Numbers next to the structures indicate the number of the cluster.


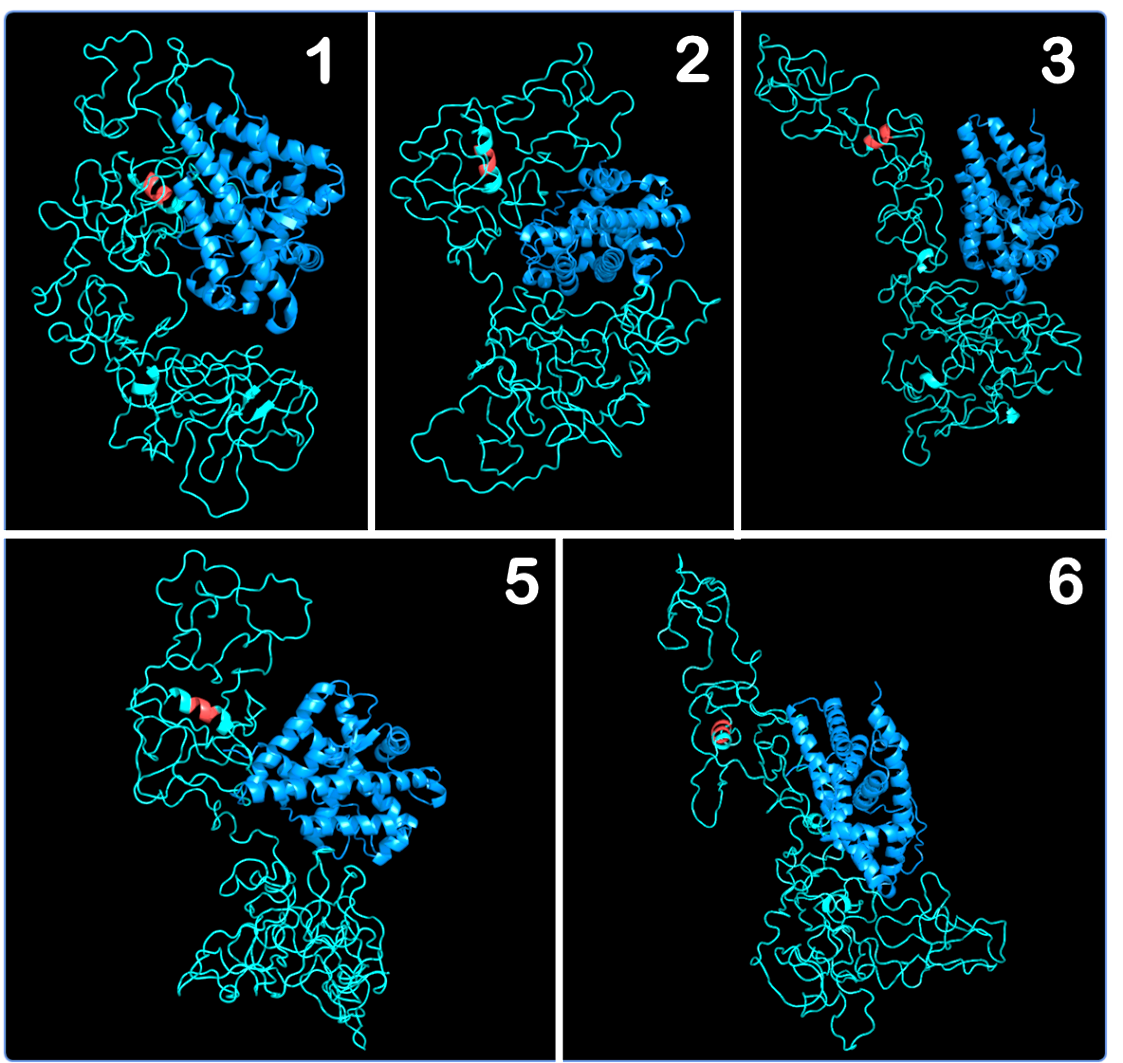


Figure S20. Representative members of each AR-NTD cluster obtained from the MD simulations in complex with AR-LBD. NTD is shown in cyan, LBD in blue and ^23^FQNLF^27^ motif in red.


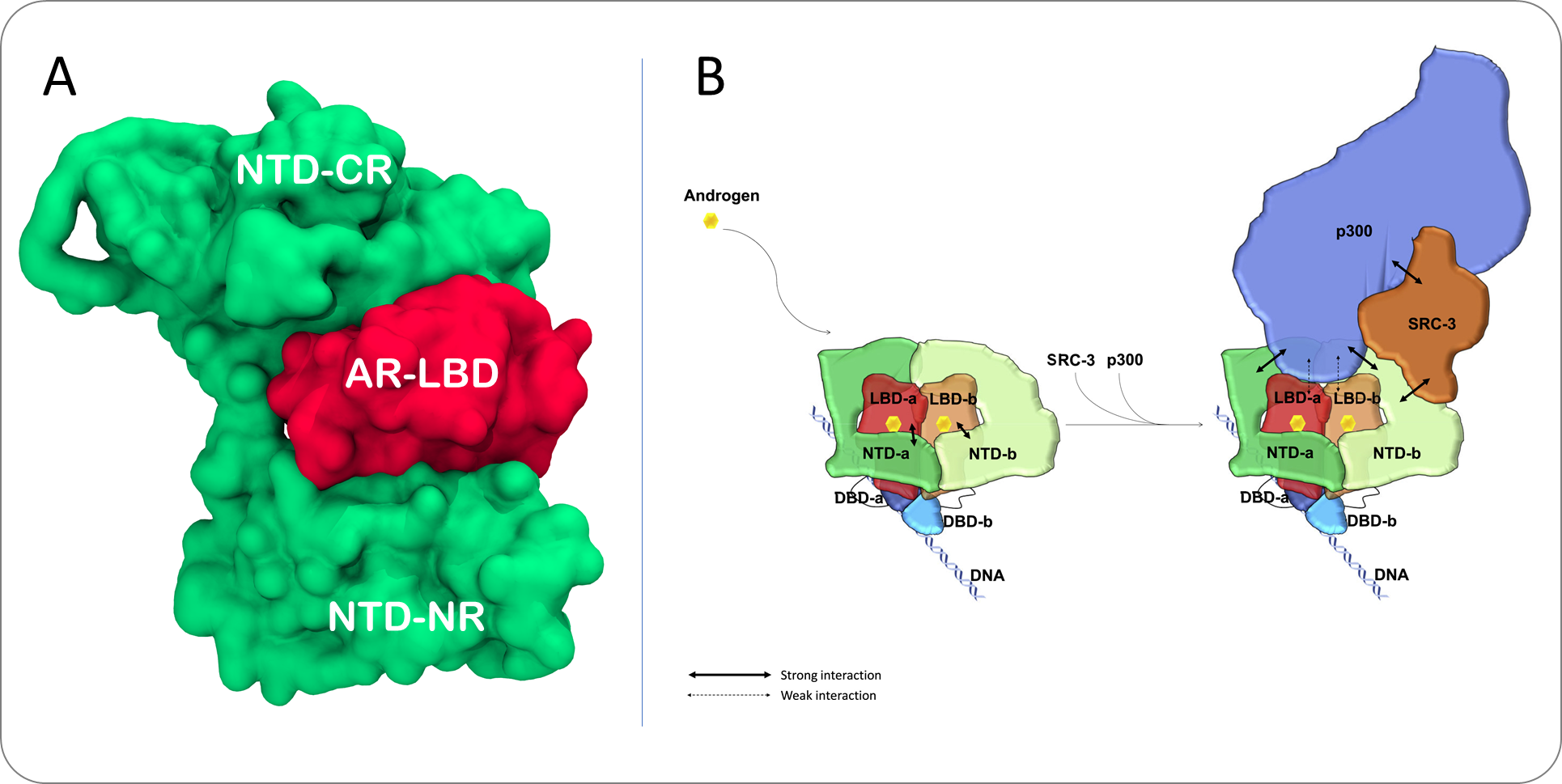


Figure S21. Comparing the model obtained from our MD simulation with the model of full-length AR assembly proposed based on Cryo-EM imaging. (A) shows surface representation of NTD (representative member of cluster 2) in complex with ligand bound AR-LBD. (B) represents model of Full-Length AR domain organization. Panel (B) is adopted with permission from [2].


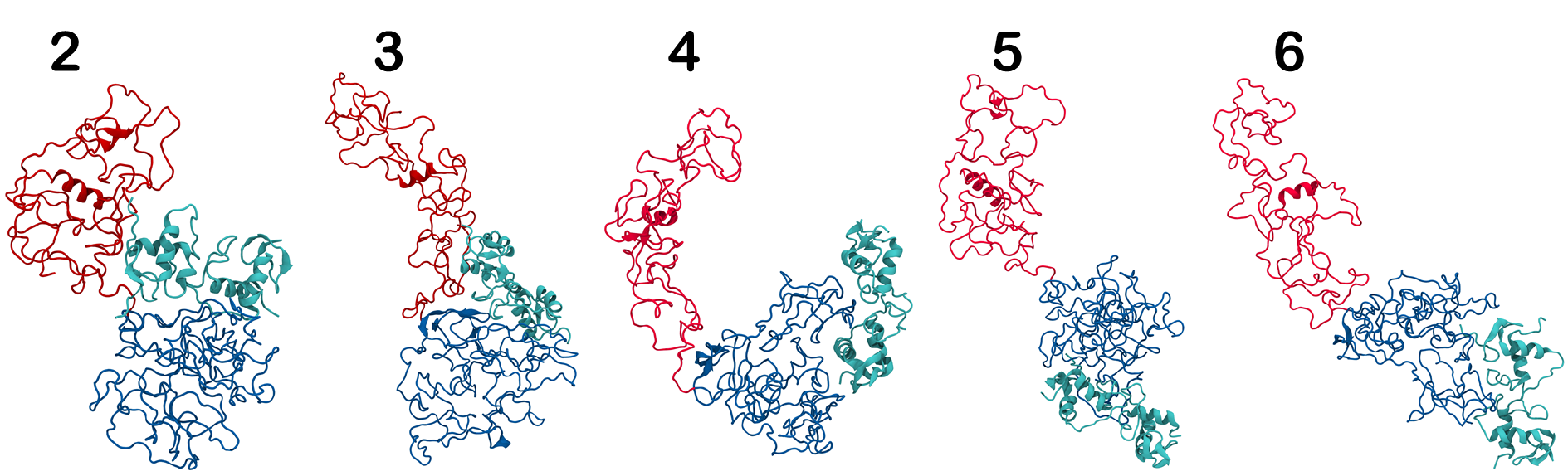


Figure S22. Representative AR-NTD structures (shown in Figure 4A) in complex with AR-DBD. CR is shown in blue, NR in red and DBD in cyan. These structures are the highest ranked poses obtained from docking analysis.


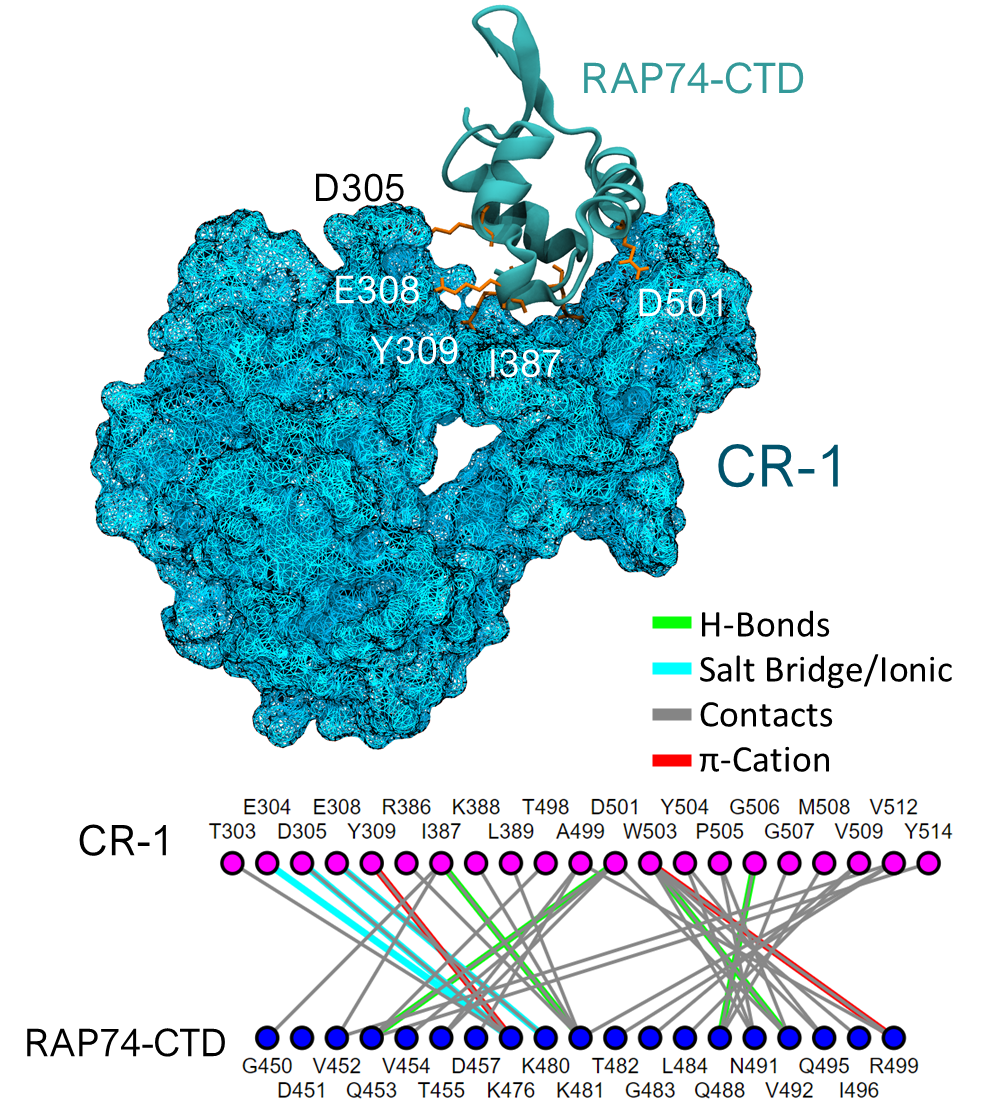


Figure S23. Representative CR structure in complex with RAP74-CTD. CR (Cluster 1) is shown in blue, RAP74-CTD in cyan. Different types of interactions are color-coded and the residue IDs are shown for the CR and RAP74-CTD regions.

**References**

[1] P. Davies, K. Watt, S.M. Kelly, C. Clark, N.C. Price, I.J. McEwan, Consequences of poly-glutamine repeat length for the conformation and folding of the androgen receptor amino-terminal domain, J. Mol. Endocrinol. 41 (2008) 301–314. https://doi.org/10.1677/JME-08-0042.

[2] X. Yu, P. Yi, R.A. Hamilton, H. Shen, M. Chen, C.E. Foulds, M.A. Mancini, S.J. Ludtke, Z. Wang, B.W. O’Malley, Structural Insights of Transcriptionally Active, Full-Length Androgen Receptor Coactivator Complexes, Mol. Cell. 79 (2020) 812-823.e4. https://doi.org/10.1016/j.molcel.2020.06.031.
